# AMPK activator ATX-304 reduces oxidative stress and improves MASLD via metabolic switching

**DOI:** 10.1172/jci.insight.179990

**Published:** 2025-04-08

**Authors:** Emanuel Holm, Isabeau Vermeulen, Saba Parween, Ana López-Pérez, Berta Cillero-Pastor, Michiel Vandenbosch, Silvia Remeseiro, Andreas Hörnblad

**Affiliations:** 1 Department of Medical and Translational Biology, Umeå University, Umeå Sweden.; 2 Maastricht MultiModal Molecular Imaging Institute (M4i), Maastricht University, Maastricht, Limburg, Netherlands.; 3 The MERLN Institute for Technology-Inspired Regenerative Medicine, Department of Cell Biology-Inspired Tissue Engineering, Maastricht University, Maastricht, Limburg, Netherlands.; 4 Wallenberg Centre for Molecular Medicine (WCMM), Umeå University, Umeå, Sweden.

**Keywords:** Gastroenterology, Hepatology, Metabolism, Fatty acid oxidation, Obesity, Transcriptomics

## Abstract

Metabolic dysfunction–associated steatotic liver disease (MASLD) is the most common chronic liver disease worldwide for which there is only one approved treatment. Adenosine monophosphate–activated protein kinase (AMPK) is an interesting therapeutic target since it acts as a central regulator of cellular metabolism. Despite efforts to target AMPK, no direct activators have yet been approved for treatment of this disease. This study investigated the effect of the AMPK activator ATX-304 in a preclinical mouse model of progressive fatty liver disease. The data demonstrated that ATX-304 diminishes body fat mass, lowers blood cholesterol levels, and mitigates general liver steatosis and the development of liver fibrosis, but with pronounced local heterogeneities. The beneficial effects of ATX-304 treatment were accompanied by a shift in the liver metabolic program, including increased fatty acid oxidation, reduced lipid synthesis, as well as remodeling of cholesterol and lipid transport. We also observed variations in lipid distribution among liver lobes in response to ATX-304, and a shift in the zonal distribution of lipid droplets upon treatment. Taken together, our data suggested that ATX-304 holds promise as a potential treatment for MASLD.

## Abstract 2

The AMPK activator ATX-304 induces changes in lipid metabolism that reduce whole-body fat mass, decrease blood cholesterol and improve fatty liver disease.

## Graphical abstract

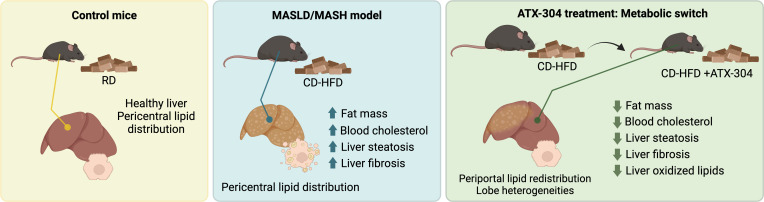

## Introduction

Metabolic dysfunction–associated steatotic liver disease (MASLD, formerly nonalcoholic fatty liver disease [NAFLD] or metabolic dysfunction–associated fatty liver disease [MAFLD]) is the most common chronic liver disease worldwide, affecting approximately one-third of the global population (1, 2). Metabolic dysfunction steatohepatitis (MASH), the severe form of MASLD, is characterized by hepatic steatosis, inflammation, liver damage, and resulting fibrosis. It is one of the leading causes of cirrhosis and end-stage liver disease, including liver cancer. The prevalence of MASH is expected to increase in the coming decade (3), paralleling the global obesity and type 2 diabetes (T2D) epidemic, thus becoming an increasingly important risk factor for liver cancer (4).

Despite the progress that has been made in understanding the etiology and pathophysiology of MASLD, there is only one drug treatment licensed for the disease. Current improvements rely primarily on lifestyle changes, including diet, exercise, and weight loss. Still, various drugs are under development with the aim to resolve MASH and achieve reduced fibrosis (5, 6). Adenosine monophosphate–activated protein kinase (AMPK) is an important protein that is involved in regulating whole-body energy homeostasis and that has received much attention as a potential target in MASLD, but also in metabolic diseases in general, including T2D. AMPK is a cellular sensor of energy status that is activated upon low energy supply such as after caloric restriction (7) or exercise (8). It has been shown that activation is stimulated by hormones involved in insulin sensitivity and fatty acid metabolism (9, 10), and that some of the positive metabolic effects of the commonly used T2D drug metformin are mediated by indirect activation of AMPK.

Upon activation, AMPK signaling adjusts cellular metabolism by inhibiting anabolic processes and promoting catabolic pathways (11). Genetic studies in mice have addressed the function of AMPK in the liver, and in the context of MASLD and MASH (12–17). Evidence shows that AMPK inhibits hepatic de novo lipogenesis and cholesterol synthesis (18), and it has also been suggested to stimulate fatty acid oxidation (17). This is to a large extent mediated by inhibition of acetyl-CoA carboxylase and HMG-CoA reductase (HMGCR), via phosphorylation (19–23). Reduced activity of AMPK is part of the pathology of MASLD, and various AMPK activators have been tested in animal models, corroborating the idea that AMPK targeting holds promise as a potential target for treatment in MASLD and MASH (13, 18, 24–26). Nevertheless, to date no direct AMPK activators have reached the market (27).

Recently, the dual AMPK and mitochondrial activator ATX-304 (formerly O304) was shown to improve glucose homeostasis and cardiovascular function in patients with T2D on metformin as well as in diet-induced obese mice (28, 29). It increases insulin sensitivity, reduces insulin resistance, improves cardiac function, and ameliorates diabetes in mouse models with impaired β cell function (30, 31). ATX-304 increases the activity of AMPK by suppressing the dephosphorylation of threonine 172 (28). Recent studies also show that ATX-304 increases cellular respiration via mitochondrial uncoupling (31). Given these data and the long-standing interest in the AMPK pathway for treatment of metabolic disease and specifically MASLD, we investigated the effect of ATX-304 treatment on mice fed a choline-deficient high-fat diet (CD-HFD) that develop progressive liver disease, including hepatic steatosis and MASH with resultant fibrosis (32). We performed transcriptomics, proteomics, spatial and bulk lipidomics analysis, as well as histological and biochemical characterization of livers from these mice. Our study demonstrates that ATX-304 reduces body fat mass, blood cholesterol, and liver steatosis, and ameliorates the development of liver fibrosis in CD-HFD mice. The effects of ATX-304 were accompanied by a shift in the metabolic program of the liver, including increased fatty acid oxidation, reduced fatty acid synthesis, and changes in cholesterol and lipid transport. ATX-304 treatment also reduced the abundance of harmful oxidized lipids in treated livers. In addition, we detected lobular heterogeneities in lipid distribution in response to ATX-304 and a consistent switch in the zonal distribution of lipid droplets upon treatment. Taken together, our data suggest that ATX-304 is a promising candidate for the treatment of MASLD, and that should be further explored for its potential clinical use.

## Results

### *ATX-304 treatment reduces whole-body fat mass and blood cholesterol of male CD-HFD mice*.

**Figure 1:**
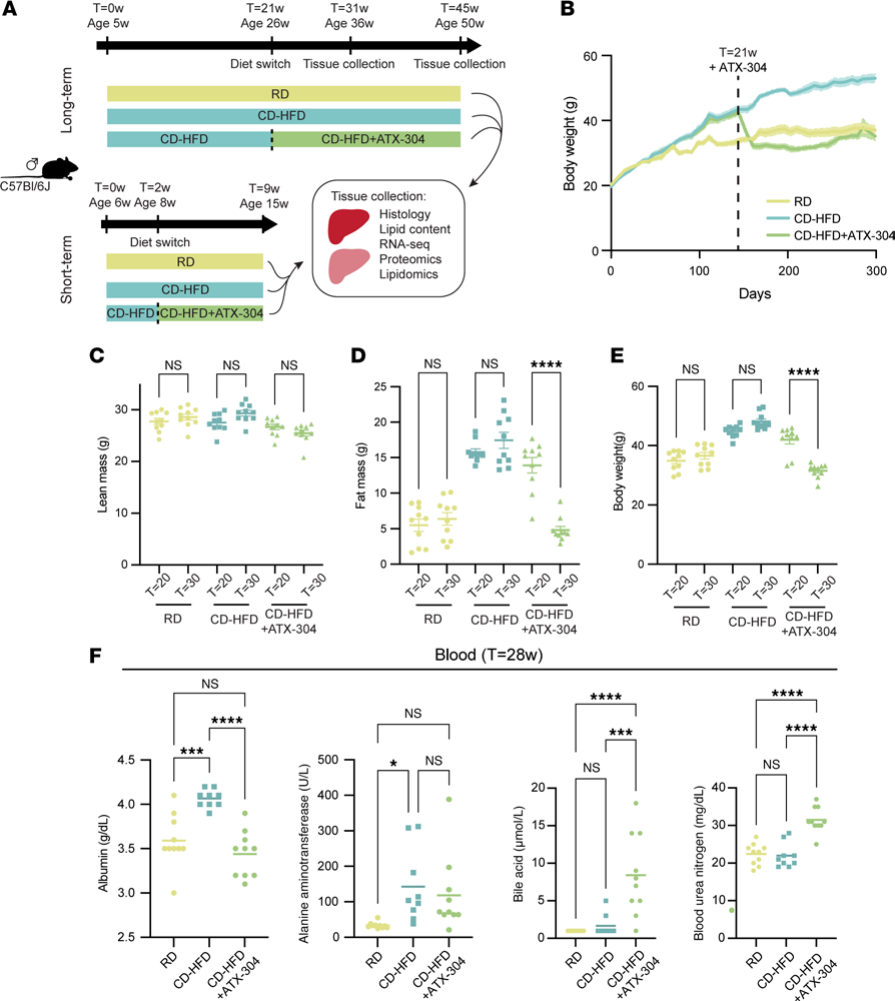
ATX-304 reduces body fat mass in male CD-HFD mice. (**A**) Overview of mouse cohorts and experimental workflow. (**B**) Weight curve for long-term RD, CD-HFD, and CD-HFD+ATX-304 mice. Dashed line indicates start of ATX-304 treatment (*T* = 21 weeks [21w]). Colored shade depicts standard error of the mean (SEM). Lean (**C**), fat (**D**), and total body mass (**E**) before (*T* = 20w) and after (*T* = 30w) ATX-304 treatment. (**F**) VetScan blood profiling of albumin (left), alanine aminotransferase, bile acid, and blood urea nitrogen for RD, CD-HFD, and CD-HFD+ATX-304 mice. *P < 0.05; ***P < 0.001; ****P < 0.0001 by 1-way ANOVA with Tukey’s multiple-comparison test. Individual data points, mean ± SEM are indicated in all graphs (*n* = 10 for all groups).

To investigate the potential positive effects of ATX-304 treatment on MASLD and MASH, 5- to 6-week-old male C57BL/6J mice were fed a CD-HFD (45% kcal fat) for 2 weeks or 21 weeks and then switched to a CD-HFD formulated with ATX-304 (1 mg/g, CD-HFD+ATX-304). The short-term cohort was treated for 7 weeks before sacrifice, while the long-term cohorts were treated for either 10 weeks or 24 weeks (Figure 1A). Control mice were fed a normal chow diet. Ingestion of CD-HFD over a longer period (21 weeks) induced liver steatosis, inflammation, and fibrosis, while the shorter period (2 weeks) represents a rather preventive setting. For all cohorts, body weight was measured regularly (Figure 1B and Supplemental Figure 1A; supplemental material available online with this article; https://doi.org/10.1172/jci.insight.179990DS1). Noticeably, within 2 weeks from the switch to the ATX-304–containing diet for the long-term experiments, the body weights of treated mice were reduced to levels similar to control mice on a regular diet (RD) (Figure 1B). Moreover, EchoMRI measurements of body composition at the start and end of the treatment demonstrated that weight reduction corresponded to a loss in fat mass, while lean weight was not reduced (Figure 1, C–E, and Supplemental Figure 1B). Still, treated mice of both short- and long-term cohorts had a slightly lower lean mass at the end of the experiment compared with RD and CD-HFD mice, demonstrating a subtle reduction in lean weight gain over time (Supplemental Figure 1C and Supplemental Figure 2). In the long-term cohort, the weight loss observed in the first 2 weeks after diet switch in the CD-HFD+ATX-304 mice was concomitant with a decrease in food intake that can partially explain the reduced fat mass (Supplemental Figure 3A). However, after the first 2 weeks, these treated mice consumed 20%–30% more food than the untreated group (Supplemental Figure 3A). For the short-term cohort, food intake was increased directly upon diet switch (Supplemental Figure 3B), and still there was a small initial decrease in body weight (Supplemental Figure 1A). Taken together, these results demonstrate that the long-term reduction in fat mass is not due to a decrease in food intake and indicates that ATX-304 mediates a metabolic switch in these mice and potentially increases energy expenditure. In line with previously published data (28, 29), ATX-304 also greatly improved glucose homeostasis and reduced insulin levels in treated mice (Supplemental Figure 4).

### *ATX-304 lowers blood cholesterol and alters cholesterol metabolism in the liver*.

**Figure 2:**
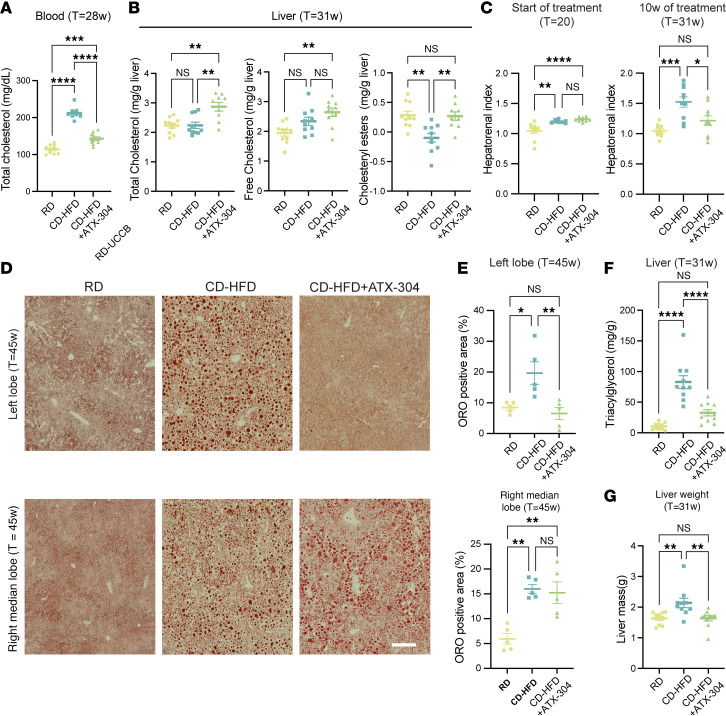
ATX-304 treatment decreases blood cholesterol and reduces liver lipid accumulation in obese CD-HFD mice. (**A**) Total blood cholesterol in RD, CD-HFD, and CD-HFD+ATX-304 mice at *T* = 28 weeks (28w) (*n* = 10 for all groups). (**B**) Graphs depicting total cholesterol (left), free cholesterol (middle), and cholesteryl ester (right) levels in livers from RD, CD-HFD, and CD-HFD+ATX-304 mice at *T* = 31w (*n* = 10 for all groups). (**C**) Hepatorenal index of RD, CD-HFD, and CD-HFD+ATX-304 mice at start (*T* = 21w) and after 10 weeks (*T* = 31w) of treatment (*n* = 10 for all groups). (**D**) ORO-stained liver sections from the left lobe (upper row) and the right median lobe (bottom row) of RD, CD-HFD, and CD-HFD+ATX-304 mice at *T* = 45w. Scale bar: 200 μm. (**E**) Percentage of ORO-positive area for the left (top) and right median (bottom) lobe is displayed in (*n* = 5 for all groups). (**F**) Liver triacylglycerol content and (**G**) liver mass for RD, CD-HFD, and CD-HFD+ATX-304 mice at *T* = 31w (*n* = 10 for all groups). *P < 0.05; **P < 0.01; ***P < 0.001; ****P < 0.0001 by 1-way ANOVA with Tukey’s multiple-comparison test. Individual data points, mean ± SEM are indicated in all graphs.

We further assessed liver function in the long-term cohort using the VetScan VS2 “mammalian liver profile” system 8 weeks after start of treatment (*T* = 28 weeks) and found that blood albumin levels were normalized in ATX-304–treated CD-HFD mice (Figure 1F), while untreated CD-HFD mice had significantly higher levels than RD controls (Figure 1F). No significant difference in the levels of alanine aminotransferase was detected between ATX-304–treated mice and untreated CD-HFD mice. However, alanine aminotransferase levels of CD-HFD+ATX-304 mice were not significantly increased compared to that of control RD mice (Figure 1F). In contrast, blood levels of both bile acids and blood urea nitrogen were elevated in ATX-304–treated animals as compared with both RD controls and untreated CD-HFD mice (Figure 1F). Moreover, blood cholesterol was almost doubled in CD-HFD mice (212 mg/dL) compared with RD control mice (114 mg/dL), while blood cholesterol was restored down to almost normal levels (143 mg/dL) in ATX-304–treated CD-HFD mice (Figure 2A).

To specifically assess the effect of ATX-304 on the livers of CD-HFD mice, animals were sacrificed after treatment and livers were collected for biochemical and histological analyses, as well as gene expression and proteomic and lipidomic profiling. Biochemical analysis of long-term cohorts at *T* = 31 weeks revealed an approximately 30% increase in total liver cholesterol in ATX-304–treated mice as compared with both healthy controls and untreated CD-HFD mice. This increase was mainly due to an increase in the free cholesterol fraction, while the amount of inactive esterified cholesterol was restored to similar levels as control livers (Figure 2B). Given the decrease in blood cholesterol levels in treated mice, the increase in hepatic cholesterol likely reflects increased hepatic uptake. In contrast, CD-HFD livers did not present a significant increase in the amount of total or free cholesterol but were devoid of cholesteryl esters (Figure 2B). Together, these data show that ATX-304 treatment improved whole-body metabolic parameters and induced weight loss via reduced fat mass. It also showed an improved blood liver profile in treated CD-HFD mice and provided evidence of an increased uptake and storage of cholesterol from the blood.

### *Heterogeneous reduction in liver lipid content and fibrosis in ATX-304–treated male CD-HFD mice*.

**Figure 3:**
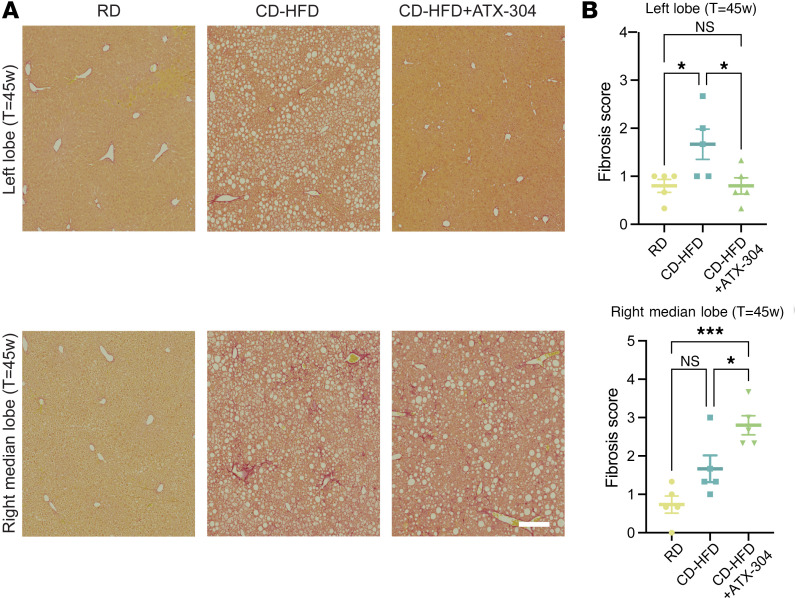
Heterogeneous amelioration of fibrosis in livers treated with ATX-304. (**A**) Representative images depicting Picrosirius red (PSR) staining of sections from the left lobe (top row) and right median lobe (bottom row) from RD, CD-HFD, and CD-HFD+ATX-304 livers. Scale bar: 200 μm. (**B**) Fibrosis score based on PSR staining for the left (top) and right median lobe (bottom) (*n* = 5 for all groups). *P < 0.05, ***P < 0.001 by 1-way ANOVA with Tukey’s multiple-comparison test.

Ultrasound analysis before the start of treatment in the long-term cohort (*T* = 20 weeks) showed that the hepatorenal index (HI) was elevated in mice on CD-HFD (HI ≈ 1.2) compared with RD controls (HI ≈ 1.0), indicating presence of hepatic steatosis (Figure 2C). After an additional 10 weeks of feeding (*T* = 30 weeks), the HI was further increased (HI ≈ 1.5) in untreated CD-HFD mice. In contrast, similar to RD controls, ATX-304–treated CD-HFD mice maintained the HI at similar levels as at the start (HI ≈ 1.0 and HI ≈ 1.2 respectively, at *T* = 31 weeks). This suggested that ATX-304 treatment suppressed liver steatosis progression in CD-HFD mice. To further assess the lipid distribution in ATX-304–treated livers, Oil Red O (ORO) staining was performed on frozen sections from the left lobe and the right median lobe for both short- and long-term cohorts (Figure 2D). These analyses, together with macroscopic examination of livers during dissection, revealed an overall reduction in lipids and lipid droplets in ATX-304–treated animals, but with substantial lobular heterogeneities (Figure 2D and Supplemental Figure 5A). In CD-HFD livers treated with ATX-304, the area of lipid droplets was normalized or even reduced as compared with RD controls in the left lobe for long-term-treated animals, while the right median lobe maintained similar levels (Figure 2E). A similar tendency in lobular distribution was evident in short-term-treated animals (Supplemental Figure 5A). Biochemical analysis also showed an approximately 60% reduction in triacylglycerol (TG) levels in livers from long-term cohorts treated for 10 weeks (Figure 2F). These histological and biochemical analyses, in conjunction with the reduced total mass of the liver (Figure 2G), demonstrate that ATX-304 treatment reduced the overall lipid content of CD-HFD livers and highlights lobular heterogeneities in lipid metabolism. In the transition from simple liver steatosis to MASH, inflammatory signaling leads to activation of hepatic stellate cells and resultant fibrosis. To evaluate the level of liver fibrosis in these mice after treatment, Picrosirius red (PSR) staining was applied to paraffin sections from the left lobe and the right median liver lobe of both short- and long-term cohorts (Figure 3A and Supplemental Figure 5C). The degree of fibrosis was significantly lower in the left lobe of ATX-304–treated compared with untreated livers and normalized to that of livers from control RD mice (Figure 3B). In contrast, the fibrosis score of tissue from the right median lobe was higher in CD-HFD+ATX-304 compared with RD and CD-HFD, showing that although there was an amelioration of fibrosis progression in the majority of the liver volume, local phenotypic differences existed between the lobes (Figure 3, A and B). A similar tendency was seen in the short-term cohort (Supplemental Figure 5, C and D). These differences correlate with the local abundance of steatosis. Taken together, these results suggest that ATX-304 treatment improves steatosis and fibrosis in male CD-HFD mice, and that these changes have a spatial pattern where the left lobe responds more strongly to the treatment regime.

### *ATX-304 mediates a transcriptional switch in liver lipid metabolism in long-term-treated CD-HFD mice*.

**Figure 4:**
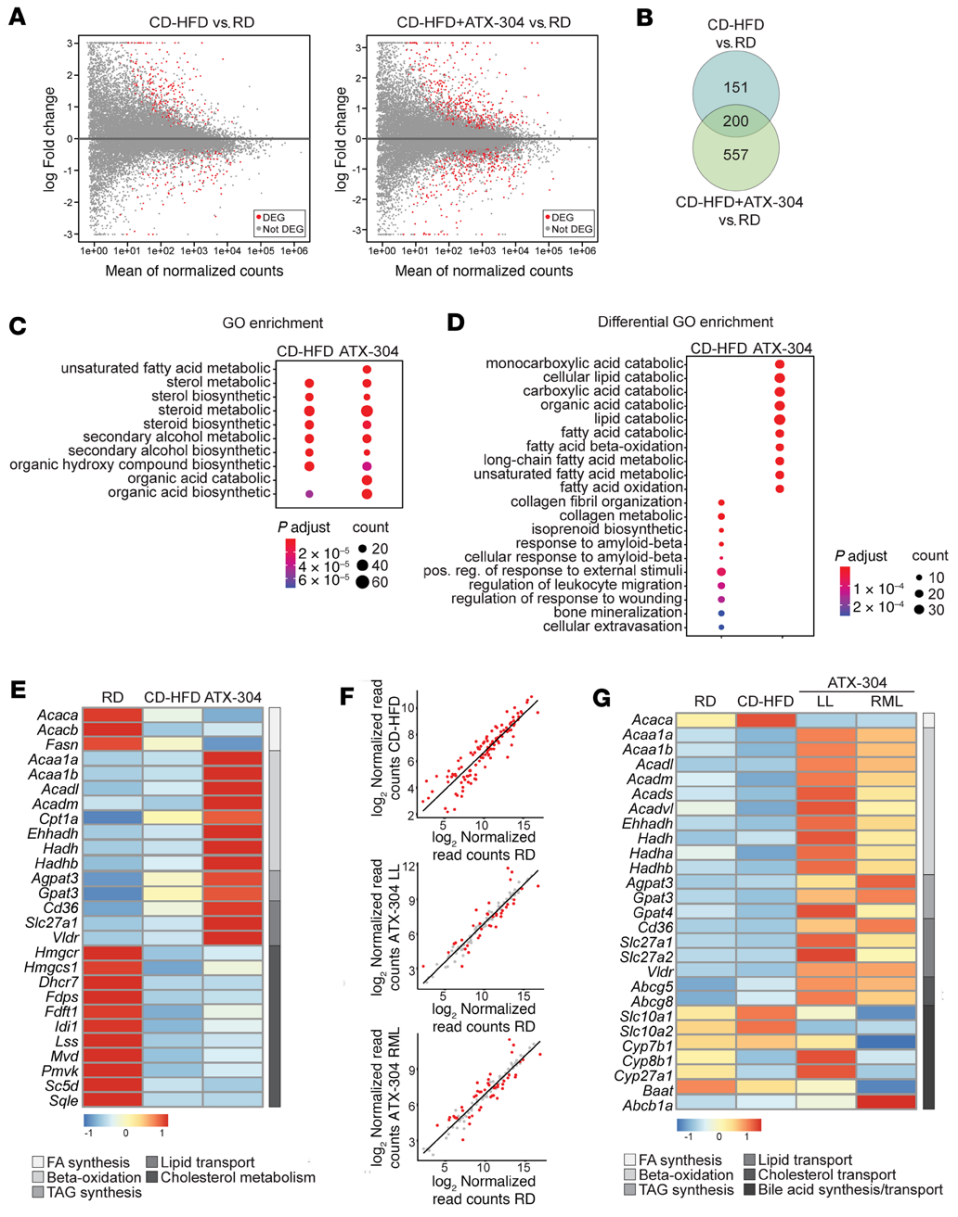
ATX-304 mediates a transcriptional switch in liver lipid metabolism. (**A**) Differentially expressed genes (DEGs, red dots) in CD-HFD (left) and CD-HFD+ATX-304 (right) compared to RD control livers (*P* < 0.01, FDR < 0.01) (**B**) Intersection of DEGs for CD-HFD and CD-HFD+ATX-304 livers. (**C**) Top 10 GO terms enriched for CD-HFD and CD-HFD+ATX-304 DEGs. (**D**) Bottom panel: Top 10 enriched GO terms unique for CD-HFD (left) or CD-HFD+ATX-304 DEGs (right). (*P* value: hypergeometric test with Benjamini’s correction). (**E**) Heatmap of normalized RNA-seq read counts for key lipid metabolism DEGs in long-term cohort at *T* = 31 weeks. Scale from blue to red indicates fold change over average read count for each row. (**F**) Gene expression levels of DEGs between left lobe of CD-HFD livers and RD livers (top) and the same genes plotted for the left lobe (LL, middle) and right median lobe (RML, bottom) of HFD-ATX-304 livers compared to the left lobe of RD liver. Red dots indicate genes that are differentially expressed in each condition (FDR < 0.01) (**G**) Heatmap of normalized RNA-seq read counts for key lipid metabolism DEGs in livers from short-term cohort at *T* = 10 weeks.

To investigate the genetic underpinnings of the observed changes in lipid content and fibrosis, RNA-seq was performed. In livers of animals fed CD-HFD for 31 weeks (*T* = 31 weeks), the number of differentially expressed genes (DEGs) compared with RD livers was 351, while for livers of CD-HFD+ATX-304 mice (*T* = 31 weeks, 10 weeks of treatment) 757 DEGs were detected (Figure 4A), Supplemental Table 1). Only 3 genes were differentially expressed between CD-HFD and CD-HFD+ATX-304 livers (Supplemental Table 1). Still, 151 DEGs were unique to CD-HFD livers and 557 DEGs were unique to ATX-304–treated CD-HFD livers (Figure 4B). Gene Ontology (GO) enrichment analysis revealed that although the most enriched GO terms were shared between CD-HFD and CD-HFD+ATX-304 (Figure 4C), ATX-304–treated livers were more associated with lipid and fatty acid catabolic processes and β-oxidation (Figure 4D). Gene expression levels of fibrogenic and hepatic stellate activation markers were increased in CD-HFD compared with RD livers. These expression levels tended to be attenuated in ATX-304–treated livers (Supplemental Figure 6), which is also in line with the histological analysis. Taking a closer look at key genes in lipid and cholesterol metabolism and annotating the uniquely expressed DEGs demonstrated that there was a shift in the transcriptional program of ATX-304–treated livers, suggesting a reduction in fatty acid synthesis and increased β-oxidation, while TG synthesis appeared to be increased. The expression of fatty acid synthesis genes (*Acaca*, *Acacb*, and *Fasn*) was reduced in ATX-304–treated livers compared with RD controls, while the expression of several genes involved in β-oxidation (*Acaa1a*, *Acaa1b*, *Acadl*, *Acadm*, *Cpt1a*, *Ehhadh*, *Hadh*, and *Hadhb*) was increased (Figure 4E). The TG synthesis genes *Agpat3* and *Gpat3* were significantly upregulated in livers of ATX-304–treated mice, which, given the strong reduction in fat mass in these animals, may reflect an increased influx of fatty acids from the blood (Figure 4E). This is also in line with the increased expression of genes encoding fatty acid transporter proteins (*Cd36*, *Slc27a1*, and *Slc27a2*) and the very-low-density lipoprotein receptor (*Vldlr*) (Figure 4E). The latter may also contribute to an increase in the influx of cholesterol into the liver, and the reduction in blood cholesterol seen in these mice (Figure 2E). Although genes involved in cholesterol synthesis were downregulated in both CD-HFD and CD-HFD+ATX-304 (Figure 4B), this difference was more pronounced in CD-HFD livers, as illustrated by some unique DEGs for these mice (*Hmgcr*, *Mvd*, *Fdft1*, and *Dhcr24*). This analysis suggests that ATX-304 mediates a catabolic switch at the transcriptional level that contributes to reduced fatty acid synthesis and increased fatty acid oxidation in the liver.

### Liver lipid metabolism is altered in short-term ATX-304–treated mice.

Given the transcriptome diversity intrinsic to the aging process (33, 34), and in view of the lobular heterogeneities in lipid distribution and fibrosis, we performed additional RNA-seq analysis for short-term-treated mice (2-week CD-HFD + 7-week CD-HFD+ATX-304, 15 weeks old). The transcriptome of the left liver lobe of RD, CD-HFD, and ATX-0304–treated CD-HFD mice, as well as the right median liver lobe from ATX-304–treated mice were analyzed. In CD-HFD livers, 115 DEGs were detected compared with RD controls. In both the left lobe and the right median lobe of ATX-304–treated livers, the expression of the majority of these genes was not significantly different from RD controls (left lobe: 78 out of 115; right median lobe: 68 out of 115) or changed in the opposite direction (left lobe: 6 out of 115; right median lobe: 9 out of 115) (Figure 4F and Supplemental Table 2). As in the long-term cohort, we detected a similar metabolic shift toward β-oxidation and lipid transport in the treated livers, albeit more pronounced with significant differences in expression levels of key enzymes also between treated and untreated animals. This was further corroborated by an observed increase in the plasma ketone levels in ATX-304–treated animals (Supplemental Figure 7). The gene expression of the fatty acid synthesis enzyme acetyl-CoA carboxylase α (*Acaca*) (Figure 4G and Supplemental Figure 8) was downregulated in both lobes of treated mice compared with CD-HFD livers, and a similar trend can be seen for acetyl-CoA carboxylase β (*Acacb*) and fatty acid synthase (*Fasn*) (Supplemental Table 2). Several β-oxidation genes were upregulated either in both lobes (*Acaa1a*, *Acaa1b*, *Acadl*, *Acadm*, *Acads*, *Ehhadh*, *Hadha*, and *Hadhb*) or in the left lobe (*Acadvl* and *Hadh*) and the expression of TG synthesis enzymes was increased in treated animals (both lobes: *Gpat3*; right median lobe: *Agpat3* and *Gpat4*) (Figure 4G and Supplemental Figure 8). We also detected significant upregulation of lipid transporter genes *Cd36*, *Slc27a1*, *Vldlr* (both lobes), and *Slc27a2* (left lobe) in treated as compared with untreated livers (Figure 4G and Supplemental Figure 8). Interestingly, cholesterol efflux transporters *Abcg5* (both lobes) and *Abcg8* (left lobe) were upregulated in ATX-304–treated compared with RD livers, although not significantly different from untreated livers, and a similar tendency was present in the long-term cohort. Also, the bile acid transporter *Slc10a2* that is involved in cholehepatic shunting was downregulated (Figure 4G), indicating a potential increase in the excretion of cholesterol and bile. This would be consistent with an accommodation for higher cholesterol uptake from the blood. No major differences in metabolic programs were found between the ATX-304–treated lobes, although it appears that the expression of β-oxidation genes might have been slightly increased in the left lobe as compared with the right median lobe (Figure 4G). Also, genes involved in bile acid synthesis (*Cyp27a1*, *Cyp7a1*, *Cyp7b1*, and *Cyp8b1*) and uptake and transport (*Slc10a1*, *Baat*, and *Abcb1a*) were differentially expressed in either the left lobe or the right median lobe of the ATX-304–treated compared with untreated livers (Figure 4G).

Taken together, these data further support the notion that ATX-304 induces a metabolic shift toward catabolism that in turn favors increased liver lipid uptake and β-oxidation, and that also appears to increase cholesterol and bile excretion. This would thus contribute to the observed amelioration of liver steatosis, suppressed progression of fibrosis, and reduction in blood cholesterol.

### Proteomics analysis corroborates catabolic switch in lipid metabolism of ATX-304–treated livers.

ATX-304 induced a clear switch in the metabolic transcriptional program of treated livers, as evidenced by RNA-seq analysis of both short-term- and long-term-treated mice. To investigate the effects also on the proteome, we performed proteomics analyses on mice treated with ATX-304 for 24 weeks (*T* = 45 weeks, long-term-treated animals). We performed liquid chromatography–tandem mass spectrometry (LC-MS/MS) analysis of tissue from the left lobe and right median liver lobe of RD, CD-HFD, and CD-HFD+ATX-304 mice. In the pairwise comparison between the experimental groups, subtle differences in the proteome were identified between the lobes but similar pathways were enriched for the left lobe or the right median lobe (Supplemental Tables 3 and 4). Thus, this analysis did not yield obvious mechanistic insight into the lobular phenotypic heterogeneities.

**Figure 5:**
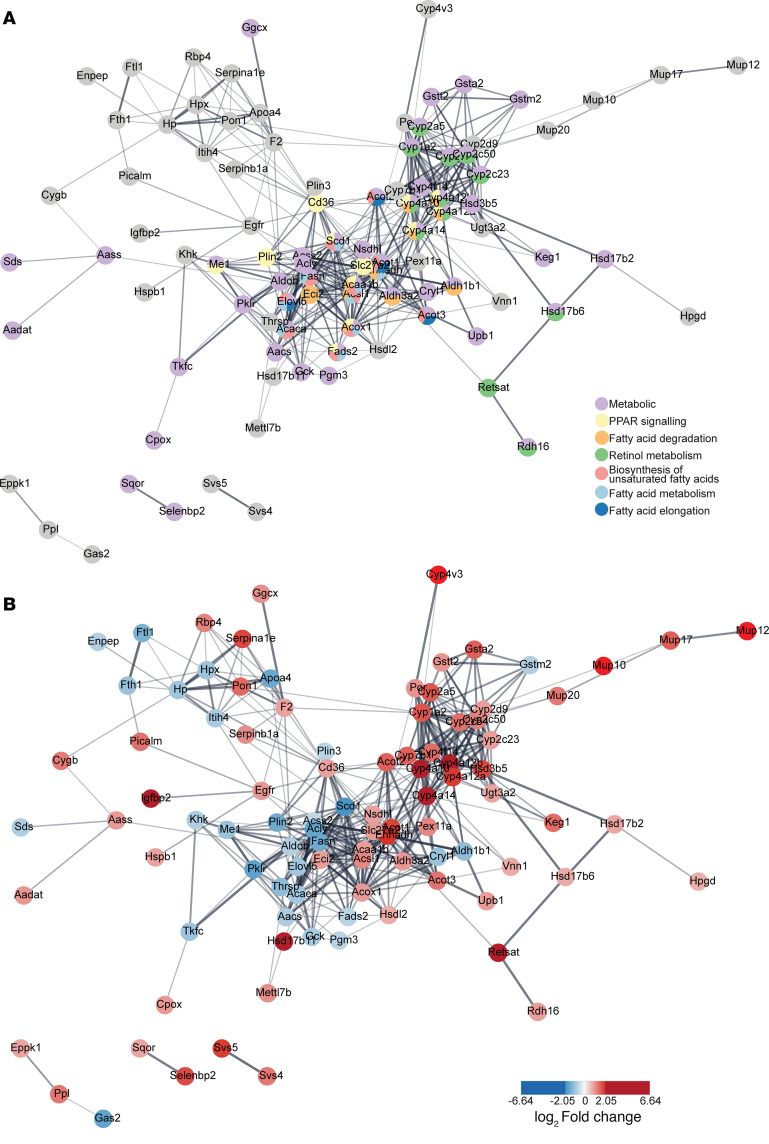
Proteomics analysis corroborates catabolic switch in lipid metabolism of ATX-304–treated livers. STRING protein interaction network of differential proteins depicting enriched KEGG pathways (**A**) and log_2_(fold change) of individual proteins (**B**).

Given the similar proteome profiles obtained from the left lobe and right median lobes, and to highlight the general proteome changes upon treatment, we considered them as whole liver. A list of 138 differential proteins between CD-HFD and CD-HFD+ATX-304 livers was extracted (Supplemental Table 5). GO analysis of differential proteins revealed a significant enrichment for terms related to fatty acid metabolism (Supplemental Table 6). In CD-HFD+ATX-304 livers compared with CD-HFD livers, abundance of proteins involved in activities such as “fatty acid hydroxylase activity,” “fatty acid hydrolase activity,” “fatty acid monooxygenase activity,” and “steroid dehydrogenase activity” were significantly increased, while proteins involved in “carboxylase and desaturase activity of co-enzymes involved in fatty acid metabolism” and “fatty acid acyl transferase related synthase activity” were significantly decreased. Similarly, Kyoto Encyclopedia of Genes and Genomes (KEGG) pathway enrichment analyses identified pathways linked to fatty acid degradation and biosynthesis, PPAR signaling, as well as metabolism (Figure 5A).

Key enzymes involved in β-oxidation (ACAA1B, EHHADH, and ACOX1) and lipid transport (CD36 and SLC27A2) were significantly increased in CD-HFD+ATX-304 livers compared with both RD and CD-HFD livers, while proteins involved in fatty acid activation (ACSL1 and ACSL4) were increased in comparison with CD-HFD–only livers (Figure 5B, Supplemental Table 5, and Supplemental Figure 9). In contrast, fatty acid synthesis enzymes ACACA and FASN were more abundant in CD-HFD compared with RD livers, with no difference between ATX-304–treated livers and RD controls (Figure 5B and Supplemental Table 5). Taken together, these data further demonstrate that ATX-304 mediates a catabolic switch that leads to reduced fatty acid synthesis and increased fatty acid oxidation in the liver. This aids in suppressing lipid accumulation in ATX-304–treated livers, despite the potential increased lipid influx as suggested also by the increased levels of lipid transporters.

### ATX-304 treatment induces remodeling of lipid zonation and ameliorates lipid profile changes in CD-HFD livers.

**Figure 6:**
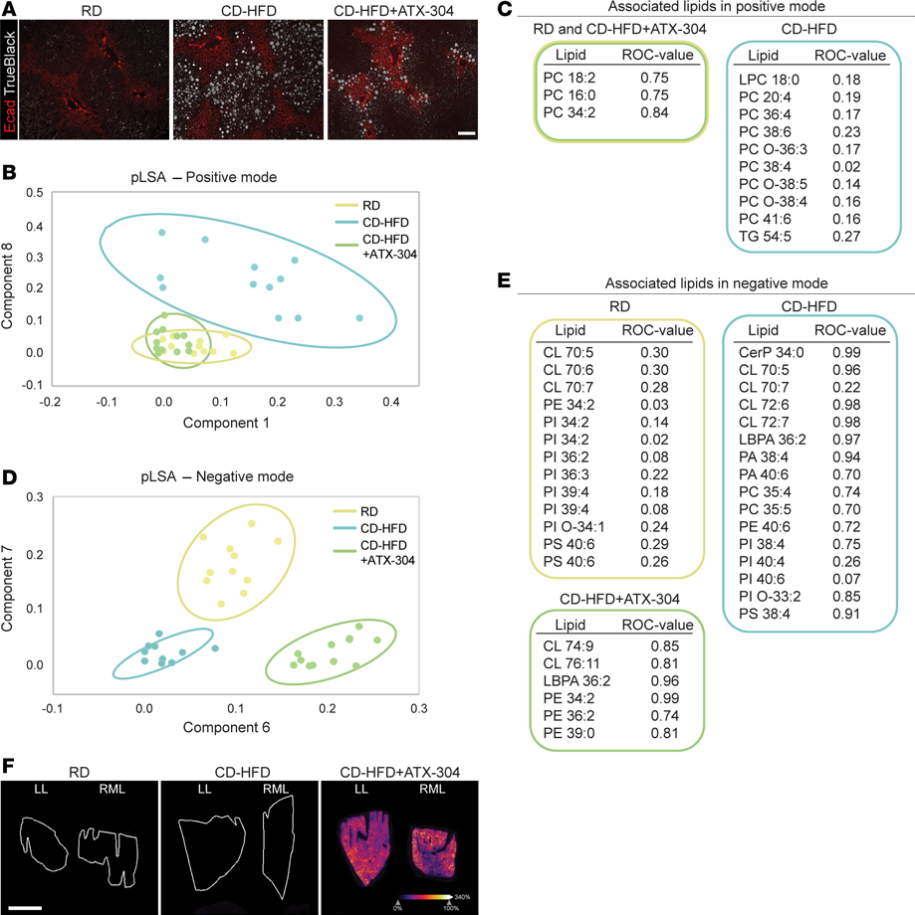
ATX-304 treatment ameliorates lipid profile changes in CD-HFD livers. (**A**) Overlay of immunofluorescence and bright-field images of liver sections stained for periportal marker E-cadherin (red) and with TrueBlack for lipid droplets (white). Lipid droplets in ATX-304–treated livers are preferentially located in the periportal zone. Scale bar: 50 μm. (**B**) pLSA score plot for all MALDI-MSI samples in positive mode. (**C**) Lipid species identified in positive mode and associated with RD and CD-HFD+ATX-304 livers (yellow and green box), or CD-HFD livers (blue box). ROC values are indicated. (**D**) pLSA score plot of all MALDI-MSI samples in negative mode. (**E**) Lipid species identified in negative mode and associated with RD (yellow box), CD-HFD (blue box), and CD-HFD+ATX-304 (green box). (**F**) Images of ATX-304 localization at 378.012 *m*/*z* ± 0.3 Da in the left lobe (LL) and right median lobe (RML) of treated animals. RD and CD-HFD livers display no signal. White scale bar: 2 mm. Graded scale from blue (0%) to white (100%) indicates signal intensity.

Histological analysis of lipid droplet distribution not only revealed reduced lipid content and lobular differences in treated livers, but also demonstrated a switch in lobular zonation pattern. As expected, both RD control livers and CD-HFD livers presented lipid droplets predominantly in the pericentral zone of the liver lobules (Figure 6A). In contrast, CD-HFD+ATX-304 animals consistently presented a higher proportion of lipid droplets toward the periportal region (Figure 6A).

It is well known that lipogenic processes such as fatty acid and TG synthesis are more active pericentrally, while β-oxidation primarily occurs periportally (35, 36). In addition, recent MS imaging has shown zonal distribution of specific lipid species and changes in these patterns upon MASDL and MASH (37, 38). To further investigate the effects of ATX-304 treatment on the lipid distribution in CD-HFD livers, we performed matrix-assisted laser desorption/ionization MS imaging (MALDI-MSI) on sections from the left and right median liver lobes of all 3 experimental groups in the long-term cohort (*T* = 45 weeks). Using this method, we were able to identify lipids of interest while preserving the spatial distribution. However, given that the lobular differences in lipid species were subtle and did not indicate specific molecular pathways underlying lobular differences (Supplemental Table 7), we combined the data to generate an overall liver lipid profile. We identified several lipid groups such as lysophosphatidylcholines (LPCs), oxidized phosphatidylcholines (oxPCs), and TGs in positive mode, while ceramides (Cers), phosphatidylethanolamines (PEs), phosphatidic acid (PA), phosphatidylinositols (PIs), phosphatidylcholines (PCs), phosphatidylserines (PSs), lysobisphosphatidic acid (LBPA), and cardiolipins (CLs) were found in negative mode.

Interestingly, unsupervised probabilistic latent semantic analysis (pLSA) of the positive-ion-mode data demonstrated that the CD-HFD livers could be clearly separated from RD controls and ATX-304–treated livers (Figure 6B). These findings indicate that the lipid profiles of the ATX-304–treated livers are more similar to RD controls and thus appear to have been partially restored. In addition, several species of oxidized lipids were characteristic of the CD-HFD samples (Figure 6C), indicating that oxidative stress may be higher in these livers compared with both ATX-304–treated livers and RD controls. Additional differences between the groups were detected in the abundance of distinct PC species, while TG 54:5 and LPC 18:0 were also more abundant in CD-HFD livers (Figure 6C). Of note, high levels of LPCs have been associated with disrupted mitochondrial integrity, inflammation, and apoptosis (39).

In negative mode, pLSA separated all 3 groups into different clusters, indicating also distinct lipid profile features for each of the groups (Figure 6B). Among the differential lipids, 15 were distinctive for CD-HFD, 12 for RD, and 6 for CD-HFD+ATX-304 livers (Figure 5E). The most abundant among differential lipids were CL (9 total) and PI (11 total), but also LBPA (2 total), PA (2 total), PC (2 total), PE (3 total), and PS (3 total) had significantly different abundance. All differential lipids obtained in positive or negative mode were further validated by receiver operating characteristic (ROC) analyses (Figure 6, C and D). All the above-mentioned lipids were homogeneously distributed across the liver lobes and did not correlate with the observed switch in zonal distribution of lipid droplets.

To further investigate the potential mechanism underlying changes in lipid droplet distribution, e.g., via differential metabolic activity due to heterogeneous uptake of ATX-304, MALDI-MSI was also employed to visualize the spatial distribution of ATX-304 in both the left lobe and right median lobe of the dosed animals (Figure 6F). These analyses revealed that the drug was evenly distributed in the livers of dosed animals, with similar high intensities between the 2 lobes (Figure 6F). As expected, no signal was found in RD and CD-HFD livers (Figure 6F). MS/MS was used to confirm the identification of the drug (Supplemental Figure 10). Thus, changes in lipid zonation were not due to variation in ATX-304 uptake, nor were the lobular heterogeneities in lipid content.

Taken together, these data demonstrate that ATX-304 treatment of CD-HFD mice leads to both quantitative and qualitative changes in liver lipid abundance, including a reduction in oxidized lipids suggestive of an amelioration of MASDL, and a shift in lipid profile toward a more normal composition.

## Discussion

The idea that AMPK holds promise as a potential target for treatment in MASLD and MASH has been around for many years. Despite efforts to test various AMPK activators in animal models, no direct AMPK activator has yet reached the clinic as treatment for this metabolic disease. Here, we investigated the effects of the clinical stage AMPK activator ATX-304 (28) in a CD-HFD mouse model of MASLD.

Our data show that ATX-304 treatment reduces whole-body fat mass, reduces hepatic steatosis, and suppresses liver fibrosis in mice fed CD-HFD. It is well documented that AMPK activation inhibits de novo lipid synthesis (13, 15, 18, 24, 40) and accumulating evidence also suggest simultaneous stimulation of fatty acid oxidation (13, 16, 18). In ATX-304–treated mice, we detected changes at the level of both gene and protein expression, in accordance with a metabolic shift in the liver favoring β-oxidation at the expense of fatty acid synthesis. In white adipose tissue, AMPK is known to inhibit lipogenesis, while the reports of effects on lipolysis are more contradictory (16, 25, 41–44). Notably, the idea that hepatic activation of AMPK triggers mobilization of fat from the adipose tissue (16) fits with several of our observations. Firstly, the almost complete reduction in whole-body fat mass suggests that ATX-304 mediates a metabolic shift toward lipid catabolism, which can also lead to an increased release of fatty acids into the circulation. Secondly, we detected upregulation of fatty acid transporters in the livers of treated animals, and zonation of lipid droplets were reversed in these mice. Like β-oxidation, evidence suggests that fatty acid trafficking occurs preferentially in the periportal zone of the liver lobules. It has been shown that there is a higher blood concentration of fatty acids in the periportal region, fatty acid binding proteins are more highly expressed (45, 46), and that glucagon induces higher uptake of fatty acids in periportal than pericentral hepatocytes (47). Although the general lipid content is reduced in livers of ATX-304–treated animals, our data suggest that in some regions with more lipids, increased periportal β-oxidation does not suffice to utilize all the fatty acids taken up from the circulation and these are used for TG synthesis. In contrast, pericentrally, lipid influx may be lower, and the combination of reduced lipogenesis and increased β-oxidation would be sufficient to reduce the abundance of lipid droplets. This balance between influx, β-oxidation, and lipid synthesis could potentially explain not only the local lobular heterogeneity in lipid abundance, but also the reversal of the lipid droplet zonation pattern seen in livers of ATX-304–treated mice. Along these lines, short-term overactivation of AMPK in the liver also leads to a concomitant initial accumulation of lipids in the liver and increased β-oxidation (48), which is also observed in the physiological response to fasting (49). Thus, these observations underline the importance of the fine-tuning between lipid utilization, lipid synthesis, and fatty acid uptake to determine phenotypic output in terms of cellular lipid content and in the long term its impact on liver function. However, our data do not indicate a clear underlying molecular mechanism for the observed heterogeneities, for which there can be several reasons, and that require in-depth high-resolution spatial studies of proteome activity, transcriptome and proteome profiles, as well as increased sample size to detect more subtle differences in molecular pathways. The lobular difference is a striking observation that also warrants further studies on whether comparable regional variations in liver lobe phenotypes are present in humans and whether this has impact on liver function and treatment, especially given that there are some reports on lobe-related differences in the progression of liver disease.

In line with previous reports that pharmacological AMPK activation reduces blood cholesterol in both rodents and primates (18), we saw a significant reduction in blood cholesterol in CD-HFD mice treated with ATX-304. Based on gene expression data and the fact that hepatic cholesterol levels are increased in these mice, it is likely that this reduction in blood cholesterol was due to increased hepatic uptake rather than reduced cholesterol synthesis in this model. Total hepatic cholesterol was more abundant in ATX-304–treated livers compared with both CD-HFD livers and controls but importantly, this increase included a normalization of esterified cholesterol levels and an increase in the ratio of esterified cholesterol to free cholesterol compared with CD-HFD. This is indicative of an improvement in the cholesterol handling capacity, as MASLD is associated with accumulation of free cholesterol without a concomitant increase in esterified cholesterol (50). The increase in blood bile acids is likely also connected to changes in the metabolic pathways of cholesterol.

Apart from reducing hepatic lipid content, ATX-304 treatment induced qualitative changes in lipid species composition in CD-HFD livers, partially restoring their lipid profile to more closely resemble that of RD control livers. More importantly, lipid profiling revealed that oxidized lipids were more abundant in livers from CD-HFD animals compared with both RD controls and ATX-304–treated livers. Oxidized lipids are formed when reactive oxygen species (ROS) react with various lipid species, and increased levels of oxidized lipids are associated with the progression of simple steatosis to MASH (51–53). Our data suggest that ATX-304–mediated activation of AMPK reduces the production of oxidized lipids in the liver, thus preventing the buildup of these harmful lipid species. This is also in agreement with other studies showing that AMPK activation mediates responses to ameliorate oxidative stress and suppresses ROS formation via induction of antioxidants and mitochondrial uncoupling (54–56). Interestingly, it was recently demonstrated that ATX-304 induces mitochondrial uncoupling in C2C12 myotubes (31). Similar mechanisms may be in place in ATX-304–treated livers and would provide an attractive hypothesis to at least partially explain how the hepatocytes cope with the increased lipid load upon activation of catabolic programs. Increased mitochondrial uncoupling led to enhanced energy expenditure, as some energy is dissipated as heat and does not contribute to ATP production. Thus, this may also explain that ATX-304–treated CD-HFD mice consumed more food than CD-HFD mice and still maintained their low fat mass after the initial reduction.

Previously studied in the context of diabetes, islet biology, and glucose homeostasis, ATX-304 is now investigated for the first time to our knowledge in a preclinical model of progressive metabolic liver disease. Our findings suggest that exploring the translational applicability of ATX-304 treatment for MASLD/nonalcoholic steatohepatitis (NASH) is warranted. In this preclinical setting, treatment with ATX-304 resulted in decreased hepatic lipid content and body adiposity, lowered blood cholesterol levels, mitigated fibrosis, and reduced oxidative stress in the liver. Furthermore, our study raises pertinent questions regarding the phenotypic and functional heterogeneity of the liver, and how regional differences may affect factors such as treatment outcomes or the accuracy of biopsy analysis. These are important considerations to take into account for future follow-up studies as well as for novel treatment regimes.

## Methods

### Sex as a biological variable

As previous studies using the same CD-HFD model primarily used male mice (32, 57, 58), we chose to also examine males given that they constitute a better characterized model and for comparison purposes.

### Animals and diets

C57BL/6J mice were purchased from Charles River Lab and housed in a 12-hour light/12-hour dark cycle in a temperature- and humidity-controlled (22°C and 50% humidity) room with ad libitum feeding. Five-week-old male C57BL/6J mice were fed either RD (Special Diet Service, 801730) or CD-HFD (Research Diets, D05010402). After 2 weeks (short-term cohort) or 21 weeks (long-term cohorts), half of the mice fed CD-HFD were switched to CD-HFD supplemented with 1 mg/g ATX-304 (CAS 1261289-04-6, provided by Betagenon AB, Umeå, Sweden). Mice in the short-term cohort were sacrificed 7 weeks after diet switch, while long-term cohorts were sacrificed after 10 or 24 weeks of treatment. Food intake and body weight were measured weekly.

### Body composition, blood profiling, and measurement of HI

Body composition (fat and lean weight) of live mice was measured using EchoMRI (EchoMRI LLC, EchoMRI 3-in-1). Blood profiling was performed on 100 μL of freshly collected blood using the Mammalian Liver Profile rotor on a VetScan VS2 (Abaxis, Inc.). Blood glucose levels were measured using a glucometer (Ultra 2, One Touch) and plasma insulin was analyzed via the ultrasensitive mouse insulin ELISA kit (Chrystal Chem Inc., 90080). Ketones were measured according to the manufacturer’s recommendations using the β-Hydroxybutyrate (Ketone Body) Assay Kit (Nordic Biosite, MET-5082). Briefly, 15 μL plasma from each animal were diluted 1:10 in 1× Assay buffer, and the solution was filtered through a 10 kDa spin filter (Sigma-Aldrich, MRCPRT010) to deproteinate samples. Samples were then loaded onto a 96-well microtiter plate in duplicate together with a standard β-hydroxybutyrate ladder. All samples were mixed with Reaction Reagent 1:1 (NAD+ cofactor 1:10, β-HB enzyme 1:200, Colorimetric Probe 1:5, in 1× Assay Buffer), followed by a 30-minute incubation in darkness at room temperature. The 96-well microtiter plate was read at 450 nm (CLARIOstar, BMG Labtech). The concentration of β-hydroxybutyrate for each sample was calculated based on the standard curve. The HI was measured using the Vevo 2100 system and the MS550D transducer (Fujifilm VisualSonics). HI was calculated as the intensity in kidney cortex echogenicity divided by the liver intensity. Three different images per mouse were used to calculate the mean.

### Liver TG and cholesterol measurements

Liver TG was measured using a Serum Triglyceride Determination Kit (Sigma-Aldrich, TR0100) according to the manufacturer’s recommendations. Briefly, 0.2–0.3 g of liver was homogenized in 2 mL PBS before addition of 6 mL chloroform/methanol (2:1). Samples were mixed until phase separation no longer occurred and left at room temperature 30 minutes before centrifuging at 4280*g* for 5 minutes. The chloroform phase was transferred into pre-weighed glassware and kept at 4°C overnight. Any water drops were removed and the chloroform evaporated by a stream of nitrogen before residual solvent was removed in a SpeedVac for 15 minutes. The glassware was re-weighed, and total lipids were calculated (mg/g liver). The residue was dissolved in 35% Triton X-100/methanol. Liver TGs were determined with the Serum Triglyceride Determination Kit according to the manufacturer’s recommendations with a minor modification for TG determination, which was analyzed at 560 nm instead of 540 nm.

Liver cholesterol was measured using the Cholesterol/Cholesteryl Ester Quantitation Kit (MBL, JM*-*K603*-*100) according to the manufacturer’s recommendations. Briefly, 25 mg of liver was homogenized in PBS. Chloroform, isopropanol, and NP-40 (7:11:0.1) were added, followed by vortexing and centrifugation at 15,000*g* for 5 minutes. The water phase was removed, and chloroform evaporated with nitrogen gas. The sample was dissolved and diluted in cholesterol assay buffer and loaded onto a 96-well plate with reaction mix (cholesterol reaction buffer, probe, enzyme mix, and cholesterol esterase). The plate was incubated in the dark for 1 hour at 37°C. Absorbance measurement was done at 570 nm using an ELISA plate reader.

### Liver histology, fibrosis scoring, and lipid droplet distribution

Liver histology and fibrosis were assessed by hematoxylin and eosin (H&E) and PSR staining on paraffin-embedded, formalin-fixed sections. Paraffin-embedded liver tissues were sectioned, stepwise deparaffinized in ethanol (100%, 95%, 70%, 50%), and rinsed with water. Mayers HTX PLUS (HistoLab, 01825) and eosin (1.5% eosin Y in 79% ethanol with 0.00025% acetic acid) were applied sequentially for 2 minutes followed by a 5-minute wash in running water in between. Slides were then rinsed in xylene and mounted with DPX mounting media (Sigma-Aldrich, 1.00579.0500) before image acquisition.

PSR was applied for 1 hour followed by 2 washes with acidified water (0.5 % glacial acetic acid in Milli-Q water). Excess water was removed, and sections were dehydrated in ethanol (2 times, 100% ethanol) followed by clearing in xylene and mounting with DPX mounting media. Fibrosis was assessed using the Ishak fibrosis scoring system (59). The mean score of 3 sections was used for each mouse (*n* = 5 for all experimental groups).

Lipid droplet distribution was assessed by ORO staining on cryo-embedded liver tissue (frozen in OCT after 4% paraformaldehyde fixation). Tissues were sectioned, dried at room temperature for 10 minutes, and washed in PBS followed by a quick rinse in 60% isopropanol. Sections were stained with ORO working solution for 15 minutes, rinsed in 60% isopropanol, and washed 2 times for 5 minutes each in Milli-Q water before mounting with Vectashield mounting media (Vector Laboratories, H-1000). Lipid content was quantified using Qupath version 0.2.2 (https://qupath.github.io/). For each sample, the mean of 3 quantified sections was used (*n* = 5 for each experimental group). All slides were imaged on an Axioscan Z1 slide scanner (ZEISS).

Immunohistochemistry on cryosectioned liver tissue was performed according to standard protocols. Sections were thawed to room temperature, rinsed in PBS, and incubated 2 hours with blocking solution (10% fetal calf serum with 0.01% sodium azide) before overnight incubation at 4°C with antibodies against E-cadherin (Invitrogen, catalog 13-900; 1:500). Primary antibodies were visualized using Alexa Fluor 594–conjugated secondary antibodies (Invitrogen, catalog A-11007; 1:1000). After immunostaining, to visualize lipid droplets, TrueBlack (Biotium) was diluted to 1× in 70% ethanol and added to slides for 30–60 seconds, followed by washing 3 times 5 minutes in PBS.

### RNA-seq

#### Library preparation.

Approximately 30 mg of frozen liver were used for total RNA extraction using the RNeasy Plus Universal minikit (Qiagen, 73404). Integrity and quality of RNA was determined on a Bioanalyzer (Agilent, 5067-1511). Poly(A) RNA was purified with the NEBNext Poly(A) mRNA Magnetic Isolation Module (NEB, E7490) and RNA-seq libraries were prepared using the NEBNext Ultra II RNA Library Prep Kit from Illumina (NEB, E7770) following the manufacturer’s instructions. The quality and concentration of libraries were assessed with a Bioanalyzer (Agilent, 5067-4626) and Qubit (Thermo Fisher Scientific, Q33230). Libraries were sequenced on a NovaSeq 6000 (Illumina) sequencer, with a coverage of approximately 37 million 150 paired-end reads per library, and quality control of fastq files was done with FastQC.

#### Gene expression analysis.

Raw reads were aligned to the mouse genome (mm10, https://genome.ucsc.edu/) using STAR (options: -- outSAMtype BAM SortedByCoordinate --seedSearchStartLmax 12 -- outFilterScoreMinOverLread 0.3 --alignSJoverhangMin 15 --outFilterMismatchNmax 33 -- outFilterMatchNminOverLread 0 --outFilterType BySJout --outSAMattributes NH HI AS NM MD --outSAMstrandField intronMotif --quantMode GeneCounts) (60). Genes with a minimum of 10 reads along all the samples were kept for further analysis. Normalization and differential expression analysis were performed using DESeq2 (61). Genes were considered differentially expressed with a *P* value of less than 0.01 and false discovery rate (FDR) less than 0.01. GO and KEGG enrichment analysis for each cluster was performed using clusterProfiler (*P* < 0.01 and FDR < 0.01) (62). Validation analysis was performed by quantitative RT-PCR using a CFX Connect Real-Time PCR detection system (Bio-Rad). All qPCR primers used are listed in Supplemental Table 8. *Ywhaz* was used as a reference gene.

### Proteomics

#### Chemicals and reagents.

Ammonium bicarbonate (ABC), dithiothreitol (DTT), iodoacetamide (IAM), formic acid (FA, ULC grade), and xylene were purchased from Sigma-Aldrich. Rapigest SF was obtained from Waters. Trypsin/Lys C was purchased from Promega. Indium tin oxide (ITO) slides were obtained from Delta Technologies. Solvents including methanol and chloroform were purchased from Biosolve Chimie SARL. For analysis of drug distribution, 1 mg of ATX-304 (Betagenon) was dissolved in 1 mL methanol (final concentration of 1 mg/mL).

#### Sample preparation for label-free proteomics.

A piece of each liver lobe was dissected and placed in 100 μL urea buffer (5 M). Tissue was homogenized 10 seconds in a minibead beater using 1.0 mm glass beads (BioSpec Products) at 2500 rpm. To lyse the cells, 3 freeze-thaw cycles were performed. Bradford assays were performed to determine protein concentration, and samples were diluted in 50 μL urea buffer (50 mM ABC/5 M urea) with a protein yield of 37.5 μg per sample. For protein extraction, 5 μL of DTT solution (200 mM) was added, followed by vortexing and incubating 45 minutes at 21°C. Then, 6 μL of IAM (400 mM) was added, samples were vortexed, and then incubated 45 minutes at 21°C in darkness. DTT solution (10 μL of 200 mM) was added to the samples, followed by vortexing and incubation for 45 minutes at 21°C. Samples were further incubated 2 hours at 37°C with trypsin/LysC in a 1:25 (1.5 μL) enzyme-to-protein ratio, followed by overnight incubation at 37°C in an Eppendorf ThermoMixer (250 rpm) upon addition of 200 μL of ABC (50 mM). The tryptic digestion reaction was stopped by addition of 30 μL of 20% ACN/10% FA. To eliminate any remaining particles, the samples were centrifuged for 30 minutes (15,000*g*, 4°C), and supernatants were collected and stored at –80°C until further processing.

#### LC-MS/MS label-free proteomics.

Separation of peptides was performed on a Thermo Fisher Scientific (Dionex) Ultimate 3000 Rapid Separation UHPLC system equipped with a PepSep C18 analytical column (15 cm, ID 75 μm, 1.9 μm Reprosil,120 Å) coupled to a Q Exactive HF mass spectrometer (Thermo Fisher Scientific). An aliquot of 2 μL (tissue piece) or 10 μL (tissue section) was injected and desalting was performed using an in-line C18 trapping column. After desalting, peptides were separated using a 90-minute linear gradient going from 5% to 35% ACN with 0.1% FA at a flow rate of 300 nL/min. Mass spectra were acquired between *m*/*z* 250 and 1250 with a resolution of 120.000 in positive polarity. This was followed by MS/MS scans in data-dependent acquisition (DDA) mode of the top 15 most intense peaks with a resolution of 15.000.

#### Data processing proteins.

Proteome Discoverer software version 2.5 (Thermo Fisher Scientific) was used to identify proteins by processing raw files with the search engine SEQUEST and the NCBI databases *Homo* *sapiens* (TaxID 9606, accessed March 25, 2020) and *Mus* *musculus* (TaxID 10090, accessed March 15, 2022). The tolerance for precursor mass was set at 10 ppm, with a fragment tolerance of 0.02 Da. Trypsin was selected as an enzyme with no more than 2 missed cleavage sites. The total peptide count was used to normalize the data. The FDR was set to a maximum of 1%. Statistical significance of protein abundance differences between CD-HFD+ATX-304 and CD-HFD livers was assessed by a 1-way ANOVA test. Proteins were considered significantly different between 2 conditions if the adjusted *P* value was less than 0.05 and the fold-change threshold was ±1.5.

#### Protein pathway analysis.

To identify the top enriched pathways, all modulated proteins were loaded into the STRING database program (https://string-db.org/, version 11.5) and EnrichR software (https://maayanlab.cloud/Enrichr/) (63–65). These pathways were considered relevant if the adjusted *P* value was less than 0.05, and they were then ordered based on their combined score in EnrichR. For pathway analysis, the KEGG database was used (https://www.genome.jp/kegg/). The pathways were visualized using the STRING database and ordered based on the calculated strength in the STRING database. This strength was calculated as the ratio of the number of proteins in our network that are annotated to the expected numbers of proteins in a random network of the same size.

### MALDI-MSI

#### Chemicals and reagents.

Norharmane was purchased from Sigma-Aldrich. ITO slides were obtained from Delta Technologies. Solvents including methanol and chloroform were purchased from Biosolve Chimie SARL. For analysis of drug distribution, 1 mg of ATX-304 (Betagenon) was dissolved in 1 mL methanol (final concentration of 1mg/mL).

#### Matrix application.

Norharmane was utilized for lipid analysis. The HTX TM-Sprayer system (HTX Technologies) was used to spray 15 layers of the matrix at a concentration of 7 mg/mL in chloroform/methanol (2:1, v/v) at a flow rate of 0.12 mL/min. The N_2_ gas pressure was set at 10 psi. A drying period of 30 seconds was employed between each layer, and a velocity of 1200 mm/min was used.

#### Data acquisition of lipids and their fragments.

For each of the 3 conditions (*n* = 3, 3 animals per condition) MALDI-MSI experiments were carried out in both polarities (positive and negative) utilizing a rapifleX MALDI TissueTyper instrument in reflectron mode (Bruker). Spectra were acquired in the mass range of *m/z* 300–1600. The molecular profiles of all conditions were compared using a spatial resolution of 30 μm. After analyzing a sample in positive mode, the same section was reanalyzed in negative mode with an offset of 15 μm in the *x* direction to avoid sampling the same location.

To structurally identify lipids, additional MALDI-MSI MS/MS experiments were performed on consecutive sections. This was done on an Orbitrap-Elite hybrid ion trap MS instrument (Thermo Fisher Scientific) running in DDA mode. MS1 data were acquired over a mass range of *m/z* 400–1600 with a resolution of 240.000 at 400 *m/z*. MS2 data were acquired in the ion trap with an isolation width of 0.7 *m/z*. In positive mode, a collision energy of 30 eV was applied, while in negative mode, a collision energy of 38 eV was used. These collision energies were fixed for the whole mass range. The stage step size was set at 25 μm (horizontal) × 50 μm (vertical) and measured in positive and negative polarity.

#### Identification of ATX-304.

MS/MS of ATX-304 was performed on a timsTOF flex (Bruker) in negative polarity. The isolation window was set at 378.00 ± 1.00 *m*/*z*. Collision energies of 20 eV and 50 eV were used. The method was used on a spotted drug standard (1 μL spotted on an ITO slide) as well as on a dosed liver section. Both were sprayed with norharmane using the same settings as mentioned above.

#### Lipid data analysis.

To compare the lipid profiles between RFD, CD-HFD, and CD-HFD+ATX-304, MALDI-MSI data were imported and analyzed in SCiLS lab software (SCiLS) (Bruker). From SCiLS, the overview spectra were exported to cvs format and imported into mMass (5.5.0, http://www.mmass.org). Afterwards, peak picking was performed in mMass with the following settings for both polarities: signal-to-noise ratio of 3, relative intensity threshold of 0.5%, and peak height of 50%. Baseline smoothing was applied with precision of 11 and an offset of 41. To exclude matrix clusters, a mass range of *m/z* 500–1600 was used. After peak picking, the spectra were imported back into SCiLS and normalized by total ion current (TIC). pLSA with random initialization was performed on the data with the *m/z* interval set to 0.3 Da for all comparisons, using the mean spectra (mean spectrum of each section separately). To validate the *m/z* values found in the pLSA, additional ROC analysis was performed with a cutoff value above 0.70 or below 0.30 for the area under the ROC curve to assign peaks as discriminative. To identify lipids species with zonal distribution, pLSA was performed on all samples by using the individual spectra (spectrum of each pixel) on the data normalized by TIC (*m/z* interval 0.3 Da) and compared to histology and immunostaining from consecutive sections. The mass range for this analysis was set to *m/z* 300–1600.

#### Data processing of lipid fragments for lipid identification.

The parent ion masses obtained from the raw full-scan Fourier transform MS were used for lipid identification with their corresponding generated ion trap–MS/MS spectra. All MS/MS files were converted into imzML and imported into LipostarMSI (66) for further analysis. The Lipid Maps database (https://www.lipidmaps.org/databases Accessed July, 2020) was used for lipid identification, such that the [M-H]^–^ ion or [M-H]^+^ ion was selected at a mass tolerance of 0.3 Da ± 5 ppm. MS/MS analyses were performed on the precursor [M-H]^–^ ion or [M-H]^+^ ion and fragments were matched with an *m*/*z* tolerance of 0.25 Da ± 5 ppm. Only lipids that had a minimum carbon chain length of 12 were used for identification in LipostarMSI.

Identification of the CLs was done manually by comparing *m*/*z* values from our high mass resolution Orbitrap data with *m*/*z* values published in the literature (67, 68). In addition, at least 2 fragments of each CL were manually annotated using the raw data. Supplemental Table 5 contains the ppm errors of all identified lipids.

### Western blotting

Whole-cell extracts were prepared from snap-frozen liver tissue from the left lobe and right median lobe in lysis buffer containing 2% SDS and 0.1 M Tris-HCl pH 6.8, 1× cOmplete Protease Inhibitor Cocktail tablet (Roche, 4693124001), and 1× phosSTOP tablet (Roche, 04906837001). Protein concentration was determined using the Pierce BCA Protein Assay Kit (Thermo Fisher Scientific) and measuring absorbance at 560 nm with the Biosan HiPo MPP-96 microplate photometer. Equal amounts of protein were run in Criterion TGX Stain-Free gels (Bio-Rad, 5678084) followed by Western blotting. Primary antibody for EHHADH was purchased from Proteintech (catalog 26570-1, 1:4000) and Peroxidase AffiniPure secondary antibody from Jackson ImmunoResearch (catalog 111-035-003, 1:10,000). For signal detection, SuperSignal West Dura Extended Duration Substrate (Thermo Fisher Scientific, 3407610220294) was used. Target protein expression was normalized to the stain-free total protein measurement using Image Lab Software (Bio-Rad).

### Statistics

Statistical tests used for each analysis are detailed in the Methods and figure legends. One-way ANOVA with Tukey’s multiple-comparison test was applied to comparisons of body weight, liver lipids, blood profiling, histological data, RT-qPCR, and Western blotting. Omics data were analyzed according to standard procedures, as described in the relevant Methods sections.

### Study approval

All experiments were performed in compliance with national and institutional laws and are reported in accordance with the ARRIVE guidelines. The study was approved by the Regional Ethics Committee at the Court of Appeal of Northern Norrland (ethical approval ID A39-2018).

### Data availability

Values for all data points in graphs are reported in the Supporting Data Values file. All data are available upon reasonable request. The RNA-seq data were uploaded to the NCBI Gene Expression Omnibus database (GEO GSE266874).

## Author contributions

The order of the first 2 authors was determined alphabetically. EH acquired, analyzed, and interpreted the data, and contributed to writing the original draft of the manuscript. IV performed lipidomics and proteomics data acquisition and analysis, and contributed to writing the original draft of the manuscript. SP contributed to tissue collection, and acquisition and analysis of histological and biochemical data. ALP generated sequencing libraries and performed bioinformatics analysis. BCP and MV supervised lipidomics and proteomics data acquisition and analysis. SR contributed to conceptualization and experimental design, and co-supervised the bioinformatics analysis. AH conceptualized and directed the research, secured funding, analyzed and interpreted the data, and wrote the final manuscript with input from EH, IV, BCP, MV, and SR.

## Supplementary Material

Supplemental data

Unedited blot and gel images

Supplemental table 1

Supplemental table 2

Supplemental table 3

Supplemental table 4

Supplemental table 5

Supplemental table 6

Supplemental table 7

Supplemental table 8

Supporting data values

## Version 1 04/08/2025

Electronic publication
