# Supplementary material for: AMPK activator ATX-304 reduces oxidative stress and improves MASLD via metabolic switching
Source: JCI Insight. 2025 Apr 8;10(7):e179990. doi: 10.1172/jci.insight.179990 (PMC11981618; doi:10.1172/jci.insight.179990)
Supplement: Supplemental data [file jciinsight-10-179990-s180.pdf]

Supplementary material

AMPK-activator ATX-304 reduces oxidative stress and improves MASLD via metabolic switching

Emanuel Holm[1*], Isabeau Vermeulen[2*], Saba Parween[1], Ana López-Pérez[1], Berta Cillero-Pastor[2,3], Michiel Vandenbosch[2], Silvia Remeseiro[1,4] & Andreas Hörnblad[1, #]

1. Department of Medical and Translational Biology, Umeå University, Johan Bures väg 12, 90187 Umeå Sweden.
2. Maastricht MultiModal Molecular Imaging Institute (M4i), Maastricht University, Maastricht, Limburg, the Netherlands
3. The MERLN Institute for Technology-Inspired Regenerative Medicine, Department of cell Biology-Inspired Tissue Engineering, Maastricht University, Maastricht, Limburg, the Netherlands
4. Wallenberg Centre for Molecular Medicine (WCMM), Umeå University, 90187 Umeå, Sweden.

* Equal contribution

\# Corresponding author: andreas.hornblad@umu.se

Author e-mails:
emanuel.holm@umu.se
i.vermeulen@maastrichtuniversity.nl
saba.parween@umu.se
ana.lopez@umu.se
b.cilleropastor@maastrichtuniversity.nl
m.vandenbosch@maastrichtuniversity.nl
silvia.remeseiro@umu.se

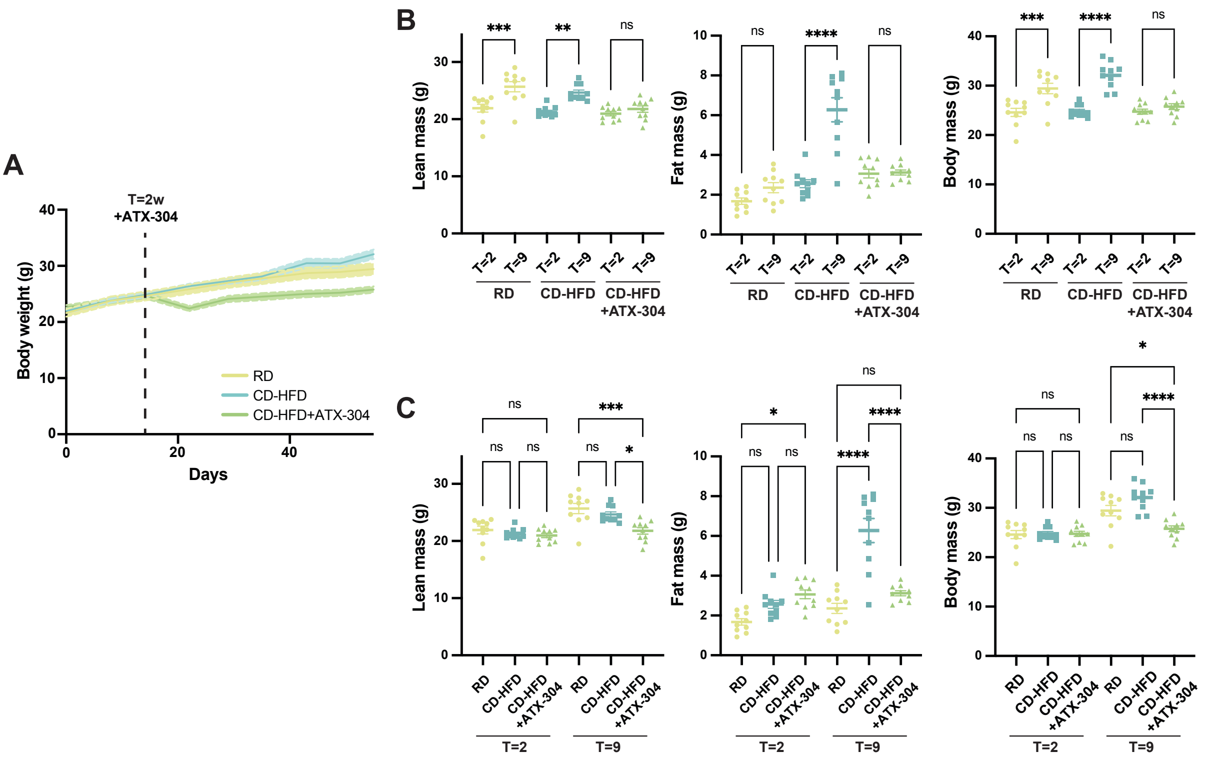

Supplementary figure 1. ATX-304 treatment reduces fat mass in CD-HFD mice. **A**) Weight curve for short-term RD, CD-HFD and CD-HFD+ATX-304 mice. Dashed line indicate start of ATX-304 treatment (T=2w). Colored shade depicts standard error of the mean (SEM). **B)** Lean, fat and total body mass in short-term cohort before (T=2w) and after (T=9w) ATX-304-treatment as measured by EchoMRI. **C**) Same data as in **B**) but depicting comparisons between RD, CD-HFD and CD-HFD+ATX-304 experimental groups before (T=2) and after treatment (T=9). **p<0.01,***p<0.001, ****p<0.0001 (One-way ANOVA with Tukey's multiple comparisons test). Individual data points, mean ± SEM are indicated (n=10 for all groups).

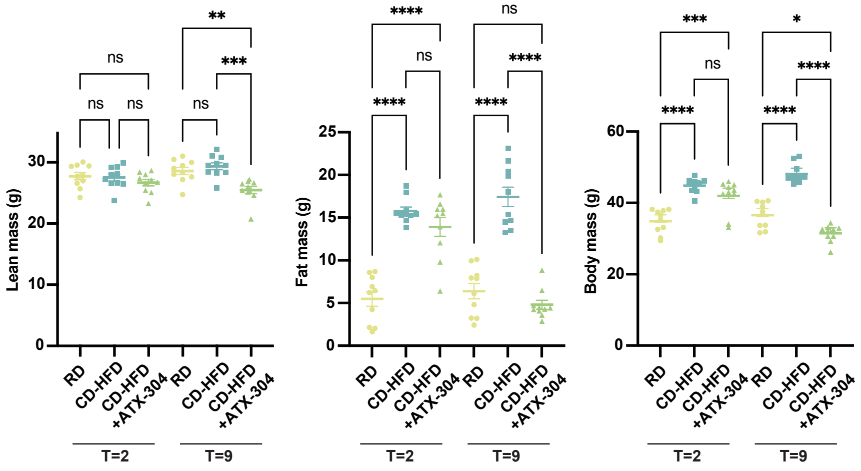

Supplementary figure 2. ATX-304 treatment reduces fat mass in CD-HFD mice. Lean, fat and total body mass as measured by EchoMRI. Same data as in Figure 1C-E but graphs depict the comparisons between RD, CD-HFD and CD-HFD+ATX-304 experimental groups in the long-term cohort before (T=20w) and after (T=30w) ATX-304-treatment . **$p<0.01$,***$p<0.001$, ****$p<0.0001$ (One-way ANOVA with Tukey's multiple comparisons test). Individual data points, mean ± SEM are indicated (n=10 for all groups).

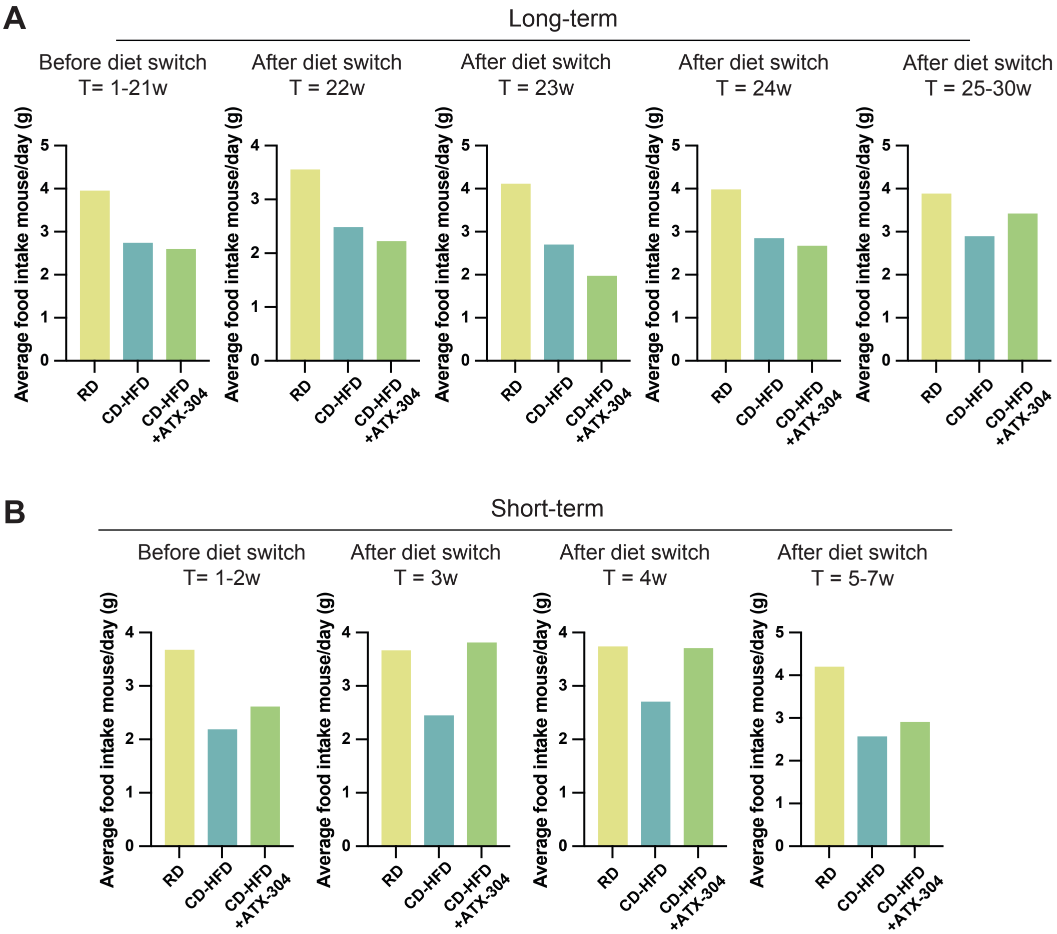

Supplementary figure 3. ATX-304 increase long-term food intake in C57Bl/6J male mice. Average food intake per day and mouse for RD, CD-HFD and CD-HFD+ATX-304 in **A**) long-term and **B**) short-term cohorts. Graphs depict the average food intake per mouse and day at indicated time periods before and after diet-switch. Average food intake is increased in both long and short-term cohorts for ATX-304-treated mice at most time points, with the exception of the initial weeks after diet switch in the long-term cohort, where food intake is reduced.

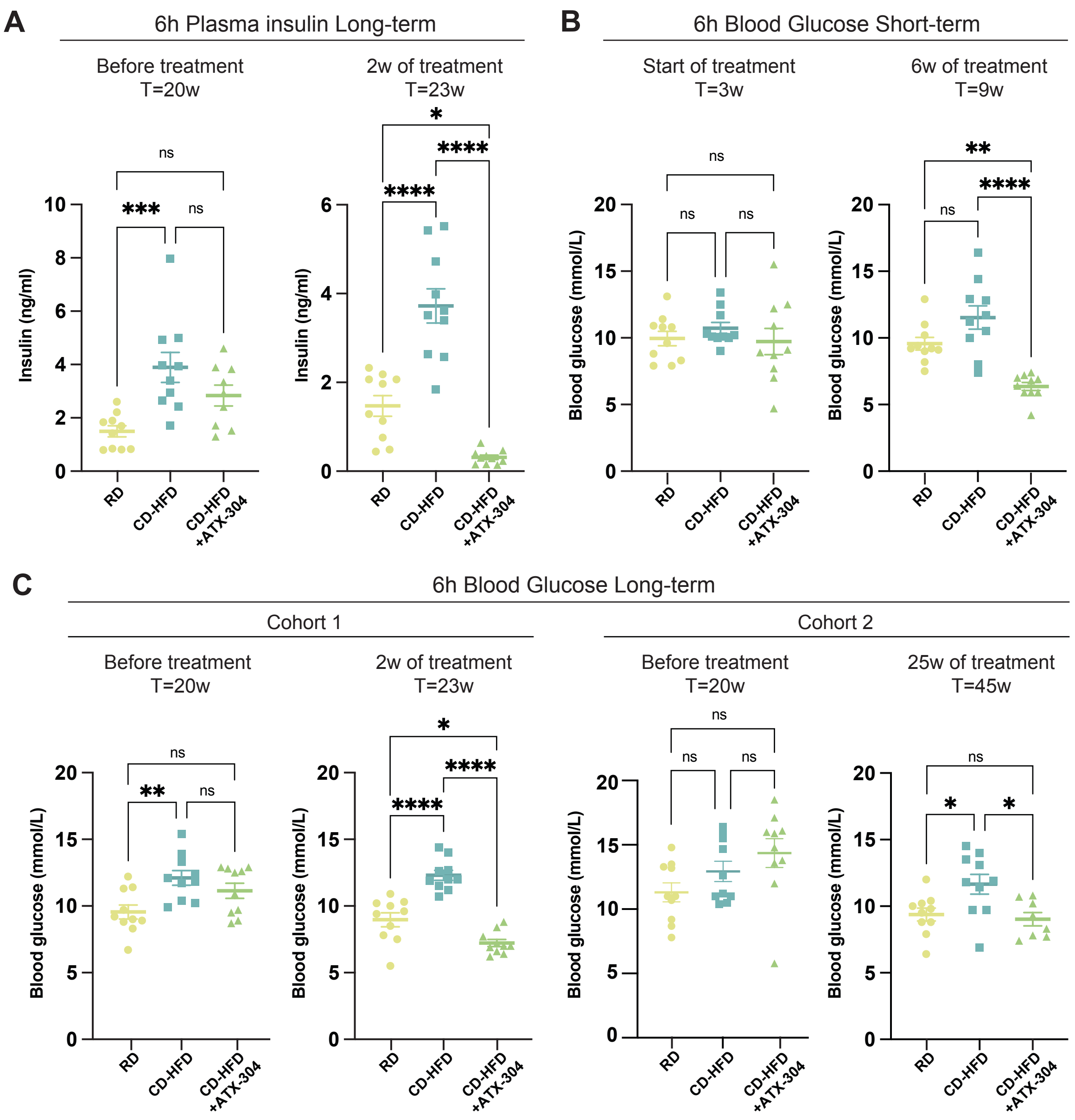

Supplementary figure 4. ATX-304 treatment significantly lowers insulin and blood glucose in C57Bl/6J male mice. A) Fasted insulin for RD, CD-HFD and CD-HFD+ATX-304 before start (T=20 weeks) and after 2w (T=23 weeks) of ATX-304 treatment. **B**) Fasted blood glucose for RD, CD-HFD and CD-HFD+ATX-304 at start (T=3 weeks) and after 6w (T=9 weeks) of ATX-304 treatment. **C**) Fasted blood glucose for RD, CD-HFD and CD-HFD+ATX-304 at start (T=20 weeks) and after 2w (T=23) or 25w (T=45w) of ATX-304 treatment. *p<0.05, ***p<0.001, ****p<0.0001 (One-way ANOVA with Tukey's multiple comparisons test). Individual data points, mean ± SEM are indicated in all graphs (n=10 for all groups).

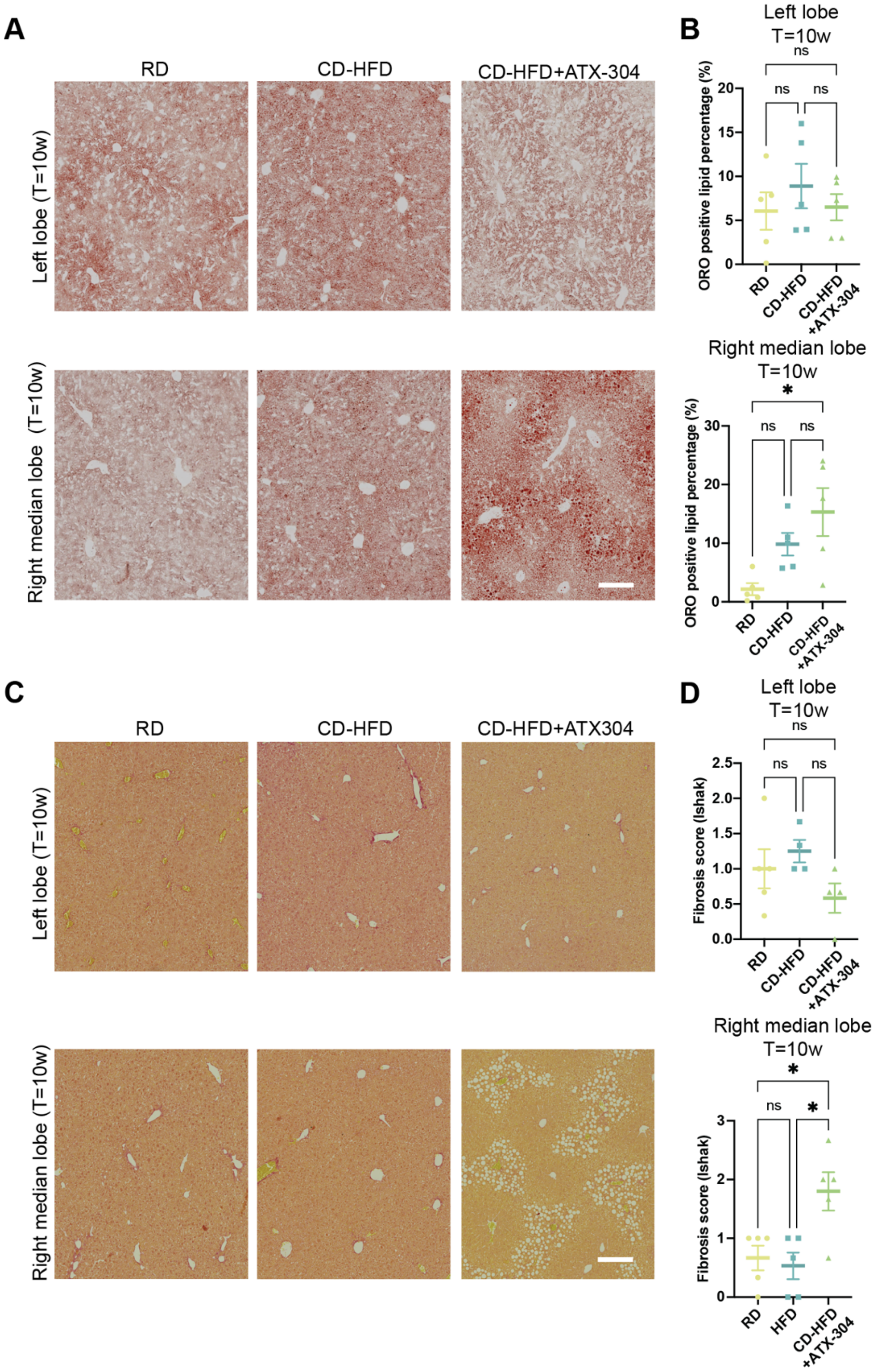

Supplementary figure 5. Lobular heterogeneities in distribution of lipids and fibrosis in ATX-304-treated livers. A) Oil red O-stained liver sections from the left lobe (upper row) and the right median lobe (bottom row) of RD, CD-HFD and ATX-304 treated mice at T=10w. Scalebar corresponds to 200um. Percentage of ORO positive area for the left (top) and right median lobe (bottom) is displayed in **B**) (n=5 for all groups). **C**) Representative images depicting picrosirius red (PSR) staining of sections from the left lobe (top row) and right median lobe (bottom row) from RD, CD-HFD and CD-HFD+ATX-304 livers at T=10w. Scalebar corresponds to 200um. **D**) Fibrosis score based on PSR staining for the left (top) and right median lobe (bottom). *p<0.05, ***p<0.001, ****p<0.0001 (One-way ANOVA with Tukey's multiple comparisons test). Individual data points, mean ± SEM are indicated in all graphs (n=5 for RD and CD-HFD, and n=4 for CD-HFD+ATX-304).

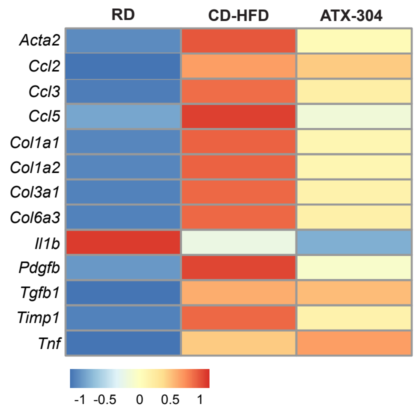

Supplementary figure 6. Expression of liver fibrosis and hepatic stellate cell activation markers in long-term treated mice. Heatmap of normalized RNA-seq read counts for markers of fibrosis and activated hepatic stellate cells in RD, CD-HFD and CD-HFD+ATX-304 from long-term cohort at T=31. Scale from blue to red indicates fold change over average read count for each row.

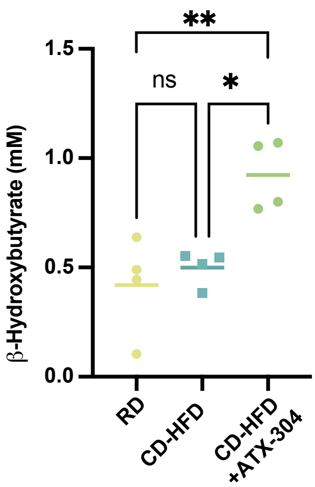

Supplementary figure 7. ATX-304 induces increased ketogenesis in short-term treated mice. Blood ß-Hydroxybutyrate levels in RD, CD-HFD and CD-HFD+ATX-304 mice at T=10. *p<0.05, **p<0.01 (One-way ANOVA with Tukey's multiple comparisons test). Individual data points, mean ± SEM are indicated in all graphs (n=4 for all groups).

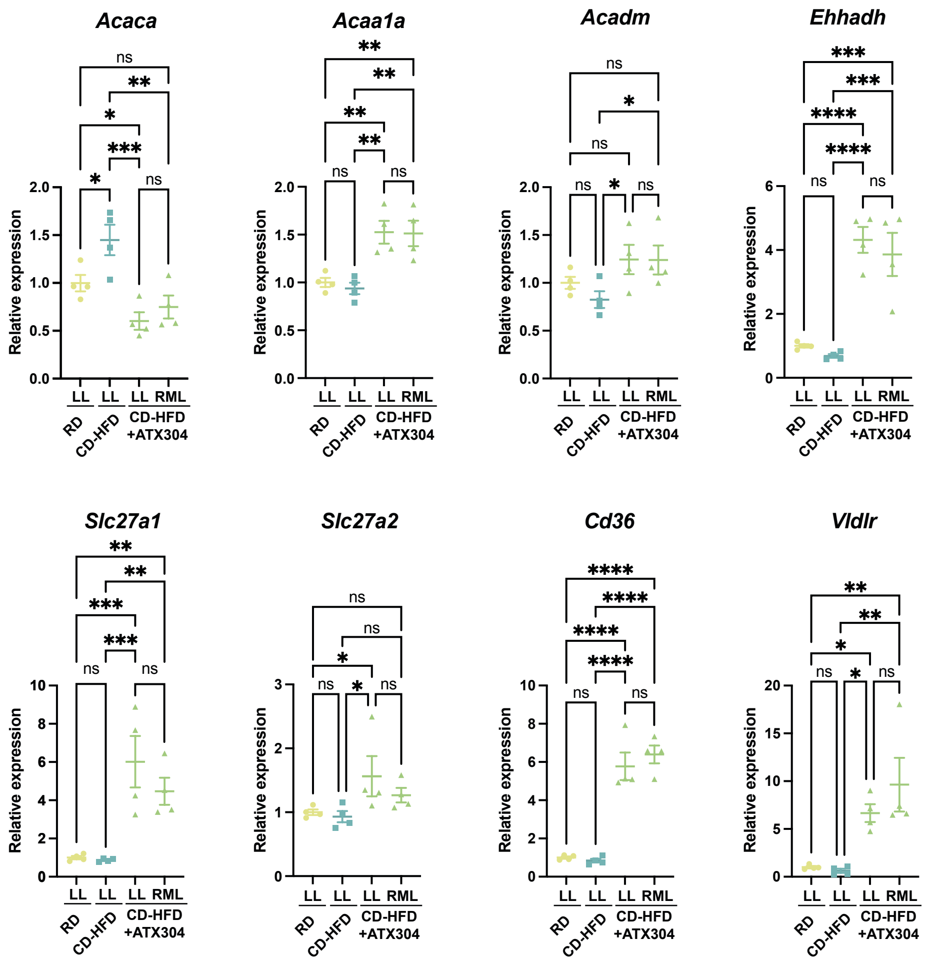

Supplementary figure 8. ATX-304 induce expression changes in liver lipid metabolism genes. Relative expression of genes involved in fatty acid synthesis (*Acaca*), beta-oxidation (*Acaa1a*, *Acadm, Ehhadh*), and lipid transport (*Slc27a1*, *Slc27a2, Cd36, Vldr*) for RD, CD-HFD and CD-HFD+ATX-304 mice at T=10. *p<0.05, **p<0.01 (One-way ANOVA with Tukey's multiple comparisons test). Individual data points, mean ± SEM are indicated in all graphs (n=4 for all groups).

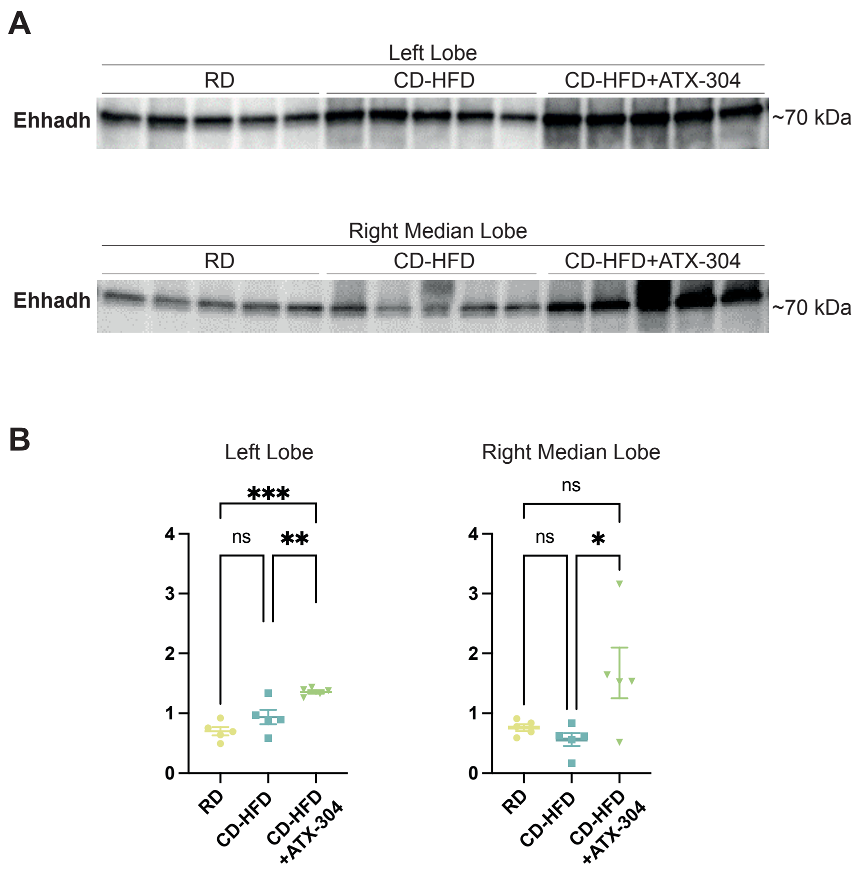

Supplementary figure 9. Protein levels of beta-oxidation enzyme EHHADH is increased in long-term treated mice. A) Immunoblot of EHHADH protein for the left lobe (upper panel) and right median lobe (lower panel) of the liver in RD, CD-HFD and CD-HFD+ATX-304 mice at T=45. **B)** Graph depicting relative protein expression normalised to total protein. *$p<0.05$, **$p<0.01$, ***$p<0.001$ (One-way ANOVA with Tukey's multiple comparisons test). Individual data points, mean ± SEM are indicated in all graphs (n=5 for all groups).

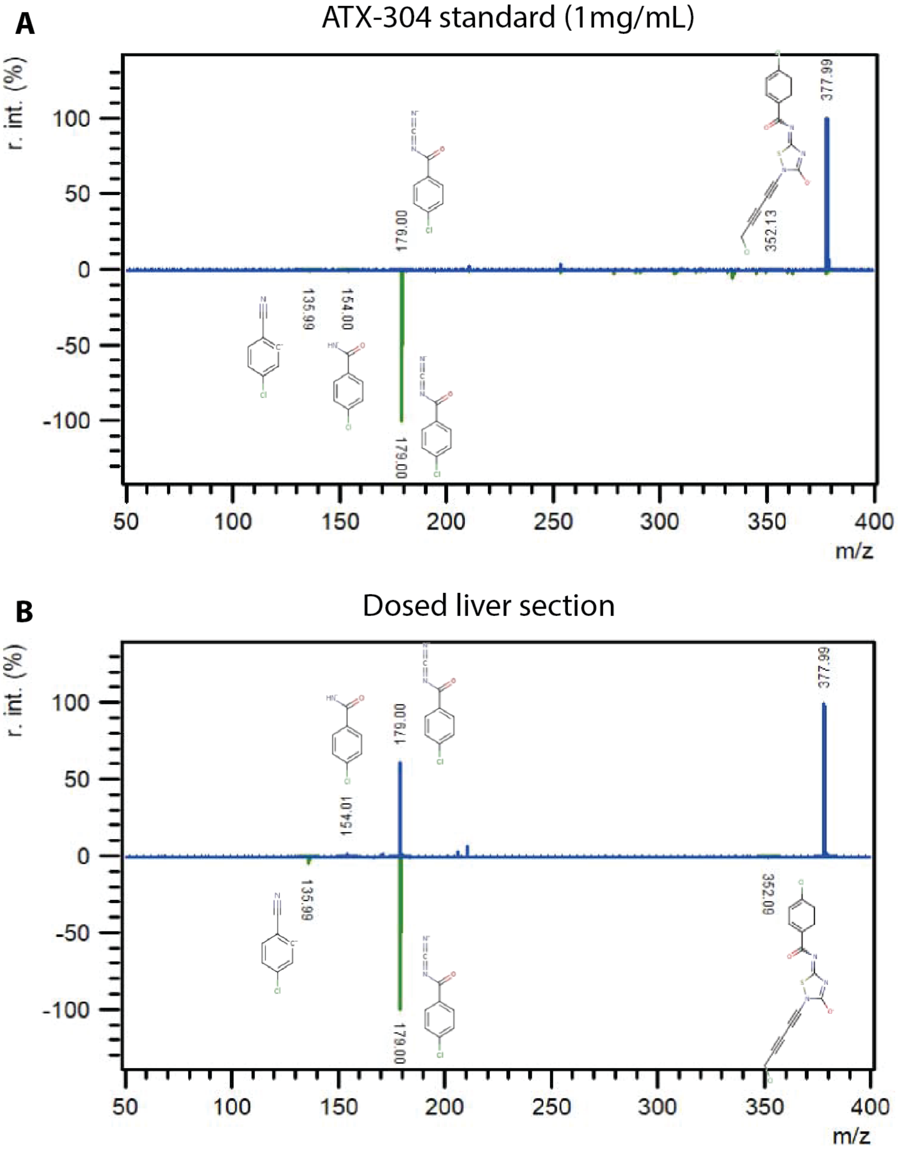

Supplementary figure 10. Identification of ATX-304 MS/MS spectrum in liver tissue sections. MS/MS spectra from **A**) ATX-304 drug standard (1mg/mL) spotted on ITO slide and **B**) dosed liver section.
