# Supplementary figures and images for: AMPK activator ATX-304 reduces oxidative stress and improves MASLD via metabolic switching
Source: JCI Insight. 2025 Apr 8;10(7):e179990. doi: 10.1172/jci.insight.179990 (PMC11981618; doi:10.1172/jci.insight.179990)

# Full unedited gels for Supplementary figure 9

## Ehhadh Left Lobe

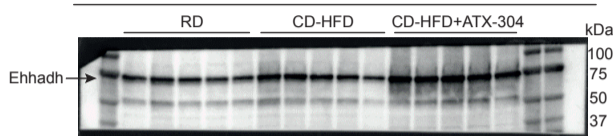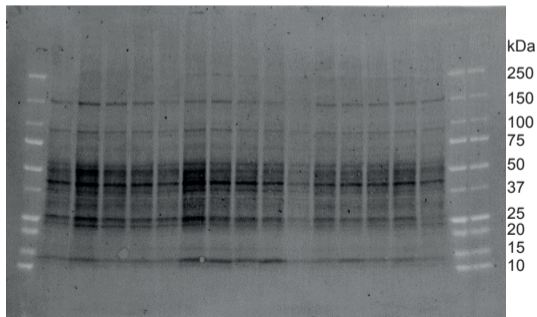

## Ehhadh Right median lobe

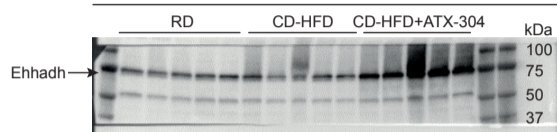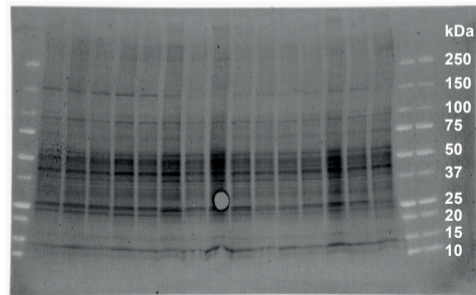

Supplement: Unedited blot and gel images [file jciinsight-10-179990-s181.pdf]
